# High-Capacity, Cooperative CO_2_ Capture in a Diamine-Appended Metal–Organic Framework through a Combined Chemisorptive and Physisorptive Mechanism

**DOI:** 10.1021/jacs.3c13381

**Published:** 2024-02-24

**Authors:** Ziting Zhu, Hsinhan Tsai, Surya T. Parker, Jung-Hoon Lee, Yuto Yabuuchi, Henry Z. H. Jiang, Yang Wang, Shuoyan Xiong, Alexander C. Forse, Bhavish Dinakar, Adrian Huang, Chaochao Dun, Phillip J. Milner, Alex Smith, Pedro Guimarães Martins, Katie R. Meihaus, Jeffrey J. Urban, Jeffrey A. Reimer, Jeffrey B. Neaton, Jeffrey R. Long

**Affiliations:** † Institute for Decarbonization Materials, University of California, Berkeley, California 94720, United States; ‡ Department of Materials Science and Engineering, University of California, Berkeley, California 94720, United States; § Department of Chemistry, University of California, Berkeley, California 94720, United States; ∥ Department of Chemical and Biomolecular Engineering, University of California, Berkeley, California 94720, United States; ⊥ Department of Physics, University of California, Berkeley, California 94720, United States; # Materials Sciences Division, Lawrence Berkeley National Laboratory, Berkeley, California 94720, United States; ∇ Molecular Foundry, Lawrence Berkeley National Laboratory, Berkeley, California 94720, United States

## Abstract

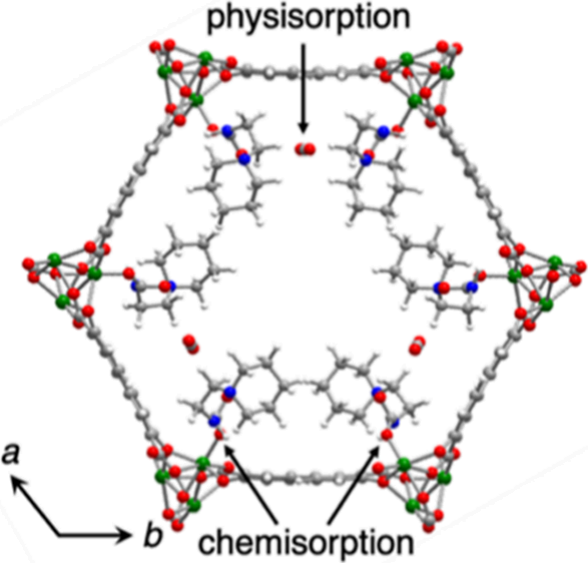

Diamine-appended Mg_2_(dobpdc) (dobpdc^4–^ = 4,4′-dioxidobiphenyl-3,3′-dicarboxylate) metal–organic frameworks are promising candidates for carbon capture that exhibit exceptional selectivities and high capacities for CO_2_. To date, CO_2_ uptake in these materials has been shown to occur predominantly via a chemisorption mechanism involving CO_2_ insertion at the amine-appended metal sites, a mechanism that limits the capacity of the material to ∼1 equiv of CO_2_ per diamine. Herein, we report a new framework, pip2–Mg_2_(dobpdc) (pip2 = 1-(2-aminoethyl)piperidine), that exhibits two-step CO_2_ uptake and achieves an unusually high CO_2_ capacity approaching 1.5 CO_2_ per diamine at saturation. Analysis of variable-pressure CO_2_ uptake in the material using solid-state nuclear magnetic resonance (NMR) spectroscopy and *in situ* diffuse reflectance infrared Fourier transform spectroscopy (DRIFTS) reveals that pip2–Mg_2_(dobpdc) captures CO_2_ via an unprecedented mechanism involving the initial insertion of CO_2_ to form ammonium carbamate chains at half of the sites in the material, followed by tandem cooperative chemisorption and physisorption. Powder X-ray diffraction analysis, supported by van der Waals-corrected density functional theory, reveals that physisorbed CO_2_ occupies a pocket formed by adjacent ammonium carbamate chains and the linker. Based on breakthrough and extended cycling experiments, pip2–Mg_2_(dobpdc) exhibits exceptional performance for CO_2_ capture under conditions relevant to the separation of CO_2_ from landfill gas. More broadly, these results highlight new opportunities for the fundamental design of diamine–Mg_2_(dobpdc) materials with even higher capacities than those predicted based on CO_2_ chemisorption alone.

## Introduction

Rising atmospheric CO_2_ levels are a leading cause of deleterious climate change, and carbon capture from point sources in the power-generation and industrial sectors is being intensively investigated as one of several key mitigation strategies.^1,2^ Aqueous amine solutions are the most mature capture technology and are used in a number of large-scale operations around the globe.^3−8^ However, these solutions exhibit extremely high regeneration energies and low oxidative and thermal stabilities and generate large amounts of waste, among other challenges, which has limited their more widespread implementation for carbon capture.^6,9−13^ To address these challenges, significant research effort has focused on the development of amine-functionalized solid sorbents such as silicas, inorganic oxides, and metal–organic frameworks as alternatives.^5,14−24^

**Figure 1:**
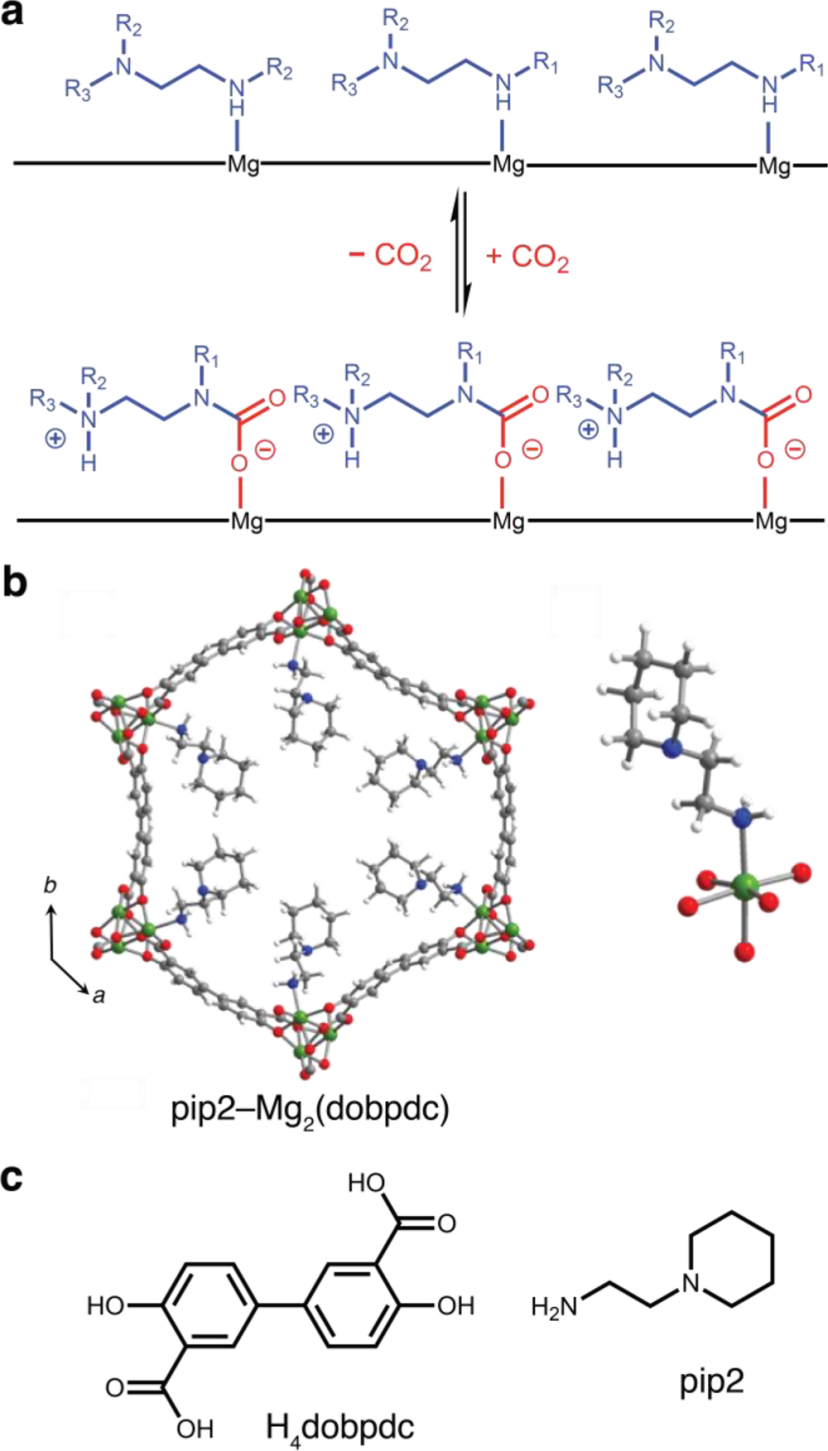
(a) Depiction of cooperative CO_2_ insertion into diamine–Mg_2_(dobpdc) to form chains of ammonium carbamate. (b) (Left) DFT-simulated structure of pip2–Mg_2_(dobpdc) and (right) detailed structure of first coordination sphere of a Mg^II^ site in this structure. Green, blue, red, gray, and white spheres represent Mg, N, O, C, and H atoms, respectively. (c) Structure of linker H_4_dobpdc (left) and pip2 (right).

Polyamine-appended frameworks of the type amine–Mg_2_(dobpdc) (dobpdc^4–^ = 4,4′-dioxidobiphenyl-3,3′-dicarboxylate),^25−35^ and recently amine–Mg_2_(olz) (olz^4–^ = (*E*)-5,5′-(diazene-1,2-diyl)bis(2-oxidobenzoate)) materials, have shown particular promise for CO_2_ capture,^36^ and some of these are being developed for testing at the pilot scale.^37^ The vast majority of these materials selectively capture CO_2_ via a cooperative mechanism involving CO_2_ insertion into the metal–amine bonds to form ammonium carbamate chains.^26^ A hallmark of this chemisorption mechanism is step-shaped CO_2_ adsorption, where CO_2_ uptake occurs within a narrow temperature or pressure window, and as a result, relatively small temperature or pressure swings can be used for material regeneration. Depending on the choice of parent framework and appended amine, these materials can exhibit very high CO_2_ capacities exceeding 3 mmol/g at a range of pressures relevant to the capture of CO_2_ from diverse flue streams. Fundamentally, the discovery of related materials exhibiting even higher CO_2_ adsorption capacities represents an important advance for the field. However, to date, the uptake of CO_2_ in diamine–Mg_2_(dobpdc) and diamine–Mg_2_(olz) materials has consistently been limited to one molecule of CO_2_ per diamine (Figure 1a), with small amounts of additional CO_2_ uptake due to physisorption.

Here, we present a new primary,tertiary (1°,3°)-diamine–appended metal–organic framework, pip2–Mg_2_(dobpdc) (pip2 = 1-(2-aminoethyl)piperidine; Figure 1b,c), that can adsorb nearly 1.5 CO_2_ molecules per appended amine, as a result of a mixed cooperative chemisorption/physisorption mechanism that is associated with a step-shaped adsorption profile.^25−32^ Together with gas sorption analysis, solid-state nuclear magnetic resonance (NMR) spectroscopy and *in situ* diffuse reflectance infrared Fourier transform spectroscopy (DRIFTS) data support a mechanism involving initial uptake of CO_2_ as ammonium carbamate, followed by simultaneous chemisorption and physisorption of CO_2_. Powder X-ray diffraction data supported by van der Waals (vdW)-corrected density functional theory (DFT) calculations indicate that CO_2_ is physisorbed in a highly ordered fashion, occupying a pocket created by ammonium carbamate chains and the framework linker. Under conditions relevant to landfill CO_2_ capture, pip2–Mg_2_(dobpdc) adsorbs nearly 5 mmol CO_2_ per gram, and extended CO_2_ cycling and breakthrough studies under simulated landfill gas^38^ reveal that this mixed adsorption mechanism is highly robust. These results highlight an opportunity to design a new family of amine-appended MOFs exhibiting enhanced CO_2_ capacities via tandem and cooperative chemisorption and physisorption for a range of capture applications.

## Results and Discussion

### Single-Component Gas Adsorption Experiments

The pip2–Mg_2_(dobpdc) material was prepared using a procedure described previously for other diamine–appended Mg_2_(dobpdc) materials.^25−32^ Briefly, methanol-solvated Mg_2_(dobpdc) (Figures S1–S3) was soaked in a toluene solution of pip2 for several hours, and then the resulting solid was isolated and activated at 130 °C under flowing N_2_ for 1 h (see Experimental Section for details and Figures S4–S6). Based on solution-phase ^1^H NMR spectroscopy analysis of a digested sample of pip2–Mg_2_(dobpdc), diamine loading in the material is quantitative (∼100%). From N_2_ adsorption data collected for activated pip2–Mg_2_(dobpdc) at 77 K (Figure S5), we calculated a Langmuir surface area of 570 m^2^/g (Brunauer–Emmett–Teller surface area = 490 m^2^/g), consistent with surface areas reported for other diamine–Mg_2_(dobpdc) variants appended with bulky diamines.^34^

**Figure 2:**
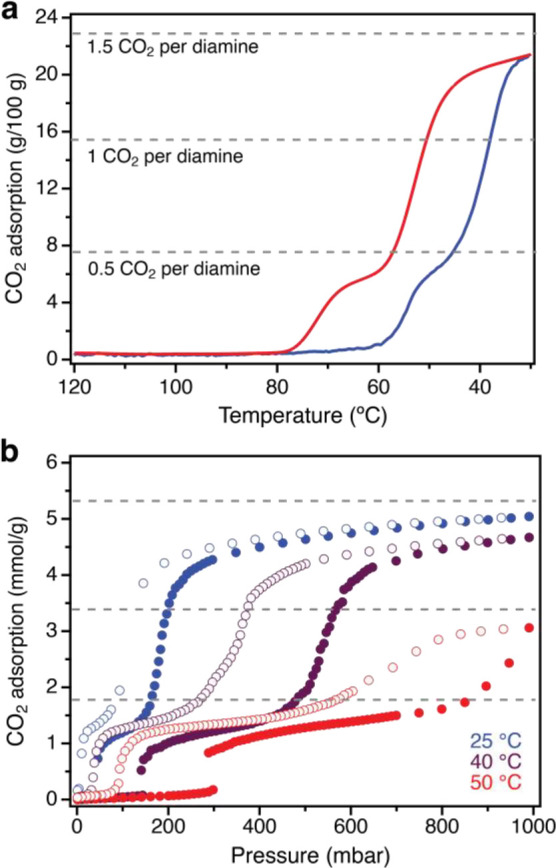
(a) Adsorption (blue) and desorption (red) isobars pip2–Mg_2_(dobpdc) under pure CO_2_ (∼1 bar), as measured by thermogravimetric analysis. (b) Pure CO_2_ adsorption isotherms for pip2–Mg_2_(dobpdc) obtained at 25, 40, and 50 °C.

As an initial assessment of the CO_2_ adsorption properties of pip2–Mg_2_(dobpdc), we collected adsorption and desorption isobars between 120 and 30 °C under 1 bar of CO_2_. The material exhibits double-step CO_2_ adsorption behavior with steps located at ∼55 and 40 °C (defined as the midpoint of the step region; Figure 2a, blue trace). Cooperative two-step CO_2_ uptake has previously been reported for several diamine–Mg_2_(dobpdc) materials featuring bulky 1°,2°-diamines,^33,34^ and it was ascribed to steric interactions between resulting ammonium carbamate chains.^34^ However, for the latter materials, each step is associated with uptake of ∼0.5 equiv of CO_2_ per diamine, and the total CO_2_ uptake in these materials has not been reported to exceed ∼1.2 CO_2_ per diamine, with the additional uptake beyond 1 CO_2_ per diamine corresponding to physisorption in the poststep uptake region.^33,34,38^ For pip2–Mg_2_(dobpdc), the first step in the adsorption isobar is also associated with uptake of close to 0.5 equiv of CO_2_ per diamine, although in contrast to previously studied materials, nearly double this quantity is taken up in the second step, resulting in an overall capacity of 1.4 CO_2_ per diamine, or 5.1 mmol/g, at 30 °C. Isobaric CO_2_ desorption from pip2–Mg_2_(dobpdc) also occurs in a two-step fashion, with moderate desorption hysteresis leading to desorption steps at ∼55 and 70 °C.

To further investigate the CO_2_ adsorption properties of pip2–Mg_2_(dobpdc), we collected the CO_2_ adsorption isotherms at 25, 40, and 50 °C (Figure 2b). The material exhibits double-step profiles at all temperatures, consistent with the isobaric experiments. At 25 and 40 °C, the first adsorption step occurs at 50 and 150 mbar, respectively, and is associated with uptake of 1.3 and 1.5 mmol of CO_2_/g; the uptake in the second step (at 200 and 500 mbar, respectively) is nearly double that of the first step (3.3 and 3.1 mmol/g, respectively), giving rise to total capacities of 4.9 and 4.6 mmol/g, respectively. This same trend is observed at 50 °C, although the uptake after each step (∼1.0 and 2.0 mmol/g, respectively) is less than at lower temperatures, consistent with the temperature-dependent uptake characterized in the adsorption isobar data. Notably, the high CO_2_ uptake achieved at 300 mbar and 25 °C (4.4 mmol/g) suggests that pip2–Mg_2_(dobpdc) may be a promising candidate for CO_2_ capture from landfill gas, which is composed of 40–60% CO_2_, 40–60% CH_4_, and 2–5% N_2_, at 25 °C (see further discussion below).^39−41^ Indeed, single-component CH_4_ and N_2_ adsorption isotherms collected for pip2–Mg_2_(dobpdc) at 25 °C suggest that the material is highly selective for CO_2_ over these other gases (Figure S7).

The CO_2_ adsorption isotherms at all temperatures were fit using linear interpolation, and the resulting fit data (pressures and loadings) were used with the Clausius–Clapeyron equation to calculate the differential enthalpy (Δ*h*_ads_) and entropy (Δ*s*_ads_) of CO_2_ adsorption as a function of loading (see Experimental Section and Figures S8–S10). From these data, we determined Δ*h*_ads_ values of −59(2) and −53(1) kJ/mol for the first and second steps, respectively, corresponding to the loading values associated with the midpoint of each step (0.5 and 2.5 mmol/g). Using the reversible heat capacity (1.29 J/g·°C) of pip2–Mg_2_(dobpdc) measured by differential scanning calorimetry (Figure S11) and the operating temperature range (adsorption at 25 °C and desorption at 80 °C), we further estimated an approximate regeneration energy of 1.58 MJ/kg CO_2_ for a temperature swing adsorption process, which is approximately one-third of the regeneration energy for a monoethanolamine solution used for landfill CO_2_ capture (∼4.5 MJ/kg CO_2_).^42,43^

### Spectroscopic Investigation of CO_2_ Uptake

**Figure 3:**
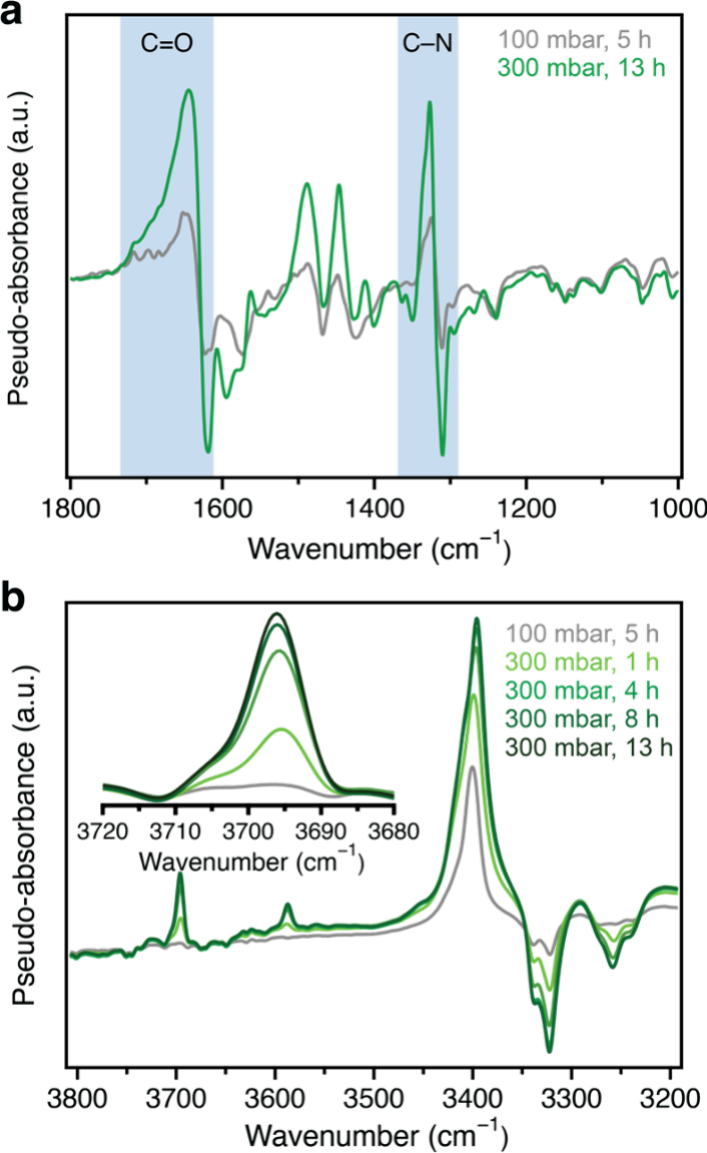
(a) Isotopic difference spectra generated by subtracting equilibrated DRIFTS data collected for pip2–Mg_2_(dobpdc) dosed with ^12^CO_2_ and ^13^CO_2_ at 100 mbar (gray trace) and 300 mbar (green trace). Blue bands highlight peaks associated with carbamate. (b) Difference spectra generated by subtracting DRIFTS data for bare pip2–Mg_2_(dobpdc) from spectra obtained for pip2–Mg_2_(dobpdc) dosed with 100 mbar of CO_2_ after 5 h and time-resolved spectra for pip2–Mg_2_(dobpdc) dosed with 300 mbar of CO_2_. Dosing with 300 mbar of CO_2_ results in the appearance of peaks at 3696 and 3587 cm^–1^ assigned to physisorbed CO_2_, concomitant with an increase in the intensity of a peak at 3394 cm^–1^ associated with carbamate formation (light to dark green).

To identify the adsorbed species formed upon CO_2_ uptake in pip2–Mg_2_(dobpdc), we collected *in situ* DRIFTS data for a sample of the material dosed with CO_2_ at room temperature and pressures of 100 and 300 mbar, corresponding to immediately after the first and second adsorption steps in the 25 °C isotherm. In separate experiments, the activated framework was first dosed with 100 mbar of natural-abundance CO_2_ (∼99% ^12^CO_2_) or ^13^CO_2_ and allowed to equilibrate before the spectra were collected (5 h in each case). Two additional experiments were carried out by dosing the activated framework with 300 mbar of CO_2_ or ^13^CO_2_, and spectra were collected at regular intervals until equilibration occurred after 13 h. Isotopic difference spectra were generated from both sets of data by subtracting the equilibrated ^13^CO_2_ spectrum obtained at 100 or 300 mbar from the corresponding equilibrated CO_2_ spectrum. As shown in Figure 3a, the two difference spectra both feature characteristic bands associated with the C=O and C–N stretches of carbamate at 1644 and 1325 cm^–1^, respectively, and the intensity of both bands increased at higher dosing pressure. These data are consistent with a mechanism involving ammonium carbamate formation upon the CO_2_ uptake.

We also generated difference spectra by subtracting the spectrum of pristine pip2–Mg_2_(dobpdc) from time-resolved spectra collected for the framework after dosing with 300 mbar of CO_2_. These spectra are plotted in Figure 3b (pale to dark green) along with a difference spectrum obtained from subtracting the spectrum for the pristine framework from the equilibrated spectrum collected after dosing with 100 mbar of CO_2_ (gray data). In the 100 mbar difference spectrum, negative peaks were apparent between 3400 and 3200 cm^–1^, along with a large positive feature at 3394 cm^–1^, consistent with the conversion of N–H vibrations associated with primary amines to those associated with secondary amines as carbamate is formed upon CO_2_ uptake. At 100 mbar, no peak was apparent for physisorbed CO_2_ (expected at ∼3696 cm^–1^; see Figure 3b, gray trace), consistent with only chemisorption occurring up to adsorption of ∼0.5 equiv of CO_2_ per diamine. However, in the time-resolved difference spectra generated after dosing with 300 mbar of CO_2_, new peaks were apparent at 3696 and 3587 cm^–1^, corresponding to the combination bands of physisorbed CO_2_.^44^ Notably, these peaks were visible within 15 min and grew concomitant with an increase in the intensity of the carbamate peak at 3394 cm^–1^, indicating that well before equilibration at this pressure, physisorption is occurring simultaneously with ammonium carbamate formation. Both peaks associated with physisorbed CO_2_ grew in intensity over the course of several hours (Figure 3b and inset) along with the carbamate peak. These data strongly suggest that CO_2_ physisorption is occurring simultaneously with chemisorption in pip2–Mg_2_(dobpdc) as the material adsorbs CO_2_ beyond 0.5 equiv per diamine.

**Figure 4:**
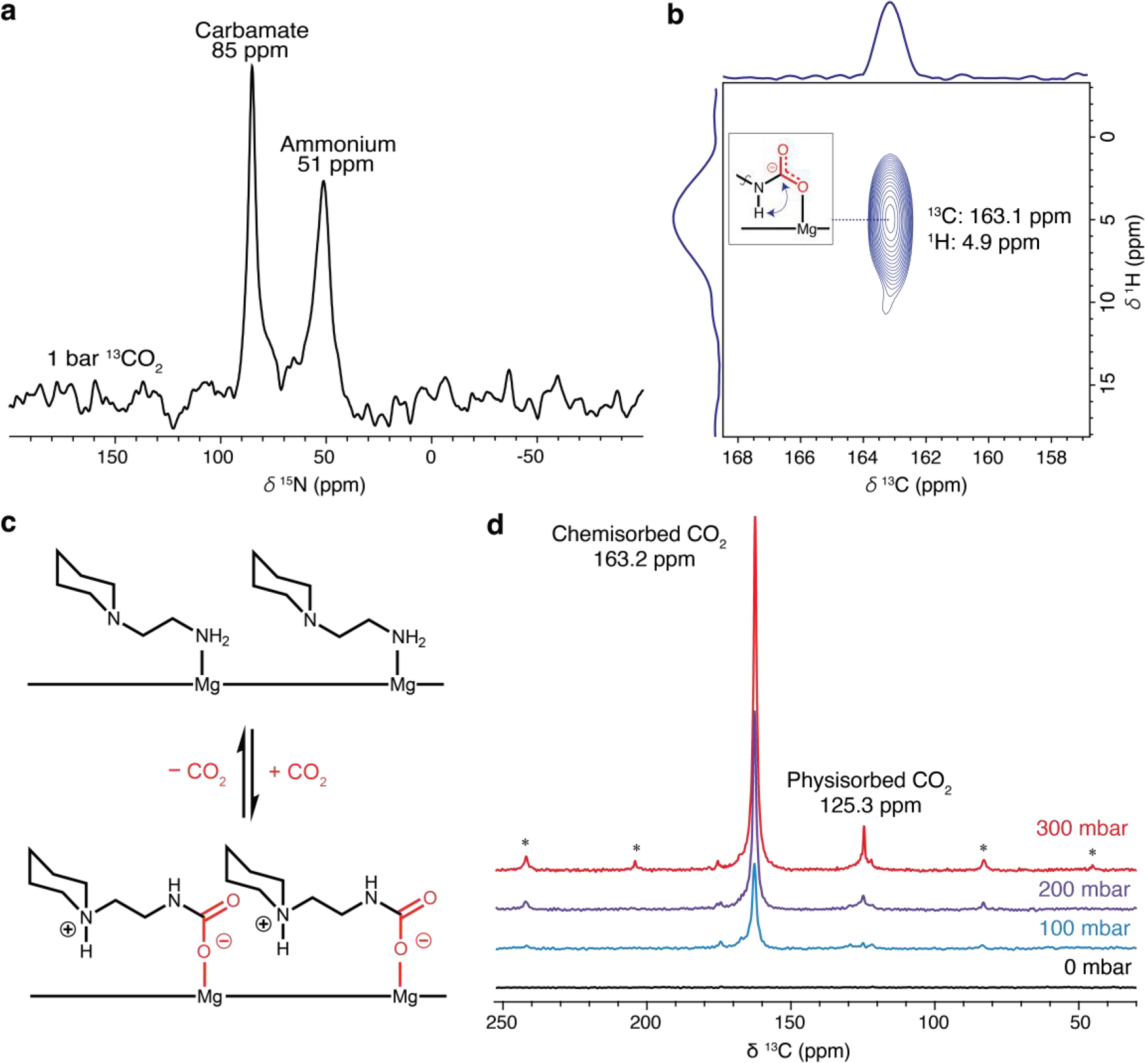
(a) Solid-state MAS ^15^N NMR spectrum (11.7 T) for pip2–Mg_2_(dobpdc) dosed with 1 bar of ^13^CO_2_, acquired by cross-polarization. (b) Solid-state ^1^H–^13^C HETCOR NMR spectrum for pip2–Mg_2_(dobpdc) dosed with 1 bar of ^13^CO_2_. (c) Illustration of the proposed mechanism for CO_2_ chemisorption in pip2–Mg_2_(dobpdc) based on solid-state NMR data. (d) Solid-state MAS ^13^C NMR spectra (11.7 T) for a sample of pip2–Mg_2_(dobpdc) dosed at different ^13^CO_2_ pressures. See Experimental Section for details.

To gain additional insight into the adsorbed species formed upon CO_2_ uptake in pip2–Mg_2_(dobpdc), we collected solid-state magic angle spinning (MAS) ^15^N and ^13^C NMR spectra after dosing the framework with ^13^CO_2_. The ^1^H → ^15^N cross-polarization spectrum of pip2–Mg_2_(dobpdc) dosed with 1 bar of ^13^CO_2_ features resonances at ∼85 and ∼51 ppm (Figure 4a). Both peaks are consistent with resonances characterized previously for carbamate and ammonium generated upon CO_2_ adsorption in various diamine–Mg_2_(dobpdc) materials.^38^ In addition, a two-dimensional ^1^H → ^13^C HETCOR spectrum collected for the same sample features a strong correlation at 4.9 ppm (^1^H) and 163.2 ppm (^13^C), consistent with the formation of ammonium carbamate species (Figure 4b,c).^38^

Solid-state MAS ^13^C NMR spectra were additionally collected for activated samples of pip2–Mg_2_(dobpdc) dosed and equilibrated at room temperature with ^13^CO_2_ pressures ranging from 0 to 300 mbar (see Experimental Section for details). After dosing with 100 mbar of ^13^CO_2_, a clear resonance was apparent at 162.7 ppm, which we assign to carbamate based on ^13^C NMR spectra reported for other diamine–Mg_2_(dobpdc) variants dosed with CO_2_.^38^ Upon increasing the ^13^CO_2_ pressure to 200 mbar, a new resonance became apparent at 125.1 ppm, which was assigned to physisorbed CO_2_.^38^ Both resonances persisted upon dosing with 300 mbar of ^13^CO_2_, consistent with the DRIFTS data. Notably, the presence of chemisorbed and physisorbed CO_2_ at 200 mbar—a pressure within the second step of the 25 °C CO_2_ isotherm—is further evidence that physisorption indeed occurs simultaneously with chemisorption in pip2–Mg_2_(dobpdc) after adsorption of an initial ∼0.5 equiv of CO_2_. Finally, integration of a ^13^C NMR spectrum collected after dosing pip2–Mg_2_(dobpdc) with 1 bar of ^13^CO_2_ (Figure S12) yielded a chemisorbed:physisorbed CO_2_ ratio of 1:0.43. Assuming saturation of the material with CO_2_ under the experimental conditions, this ratio is consistent with the total uptake of ∼1.4 equiv of CO_2_ per diamine in pip2–Mg_2_(dobpdc) (Figure 2b), significantly more than the 10–15% physisorbed CO_2_ that has been reported for other diamine-appended frameworks.^38^ Altogether, these results indicate that the unusually high CO_2_ capacity of pip2–Mg_2_(dobpdc) arises due to a unique cooperative mechanism involving the simultaneous physisorption of CO_2_ with CO_2_ chemisorption at pressures corresponding to the second isothermal adsorption step. In contrast, for other diamine-appended frameworks, chemisorption alone is associated with the step regions of the isotherm, whereas physisorption is predominantly a poststep phenomenon.^33,34^

### Structural and Computational Investigation of CO_2_ Binding

To elucidate the structure resulting upon CO_2_ uptake in pip2–Mg_2_(dobpdc) and identify the location of the physisorbed CO_2_, we collected *in situ* powder X-ray diffraction data for a sample of the framework before and after dosing with 1 bar of CO_2_ at 298 K (see Experimental Section for details). The diffraction pattern of pip2–Mg_2_(dobpdc) features more peaks than that of Mg_2_(dobpdc) (Figure S1), for example, three low intensity peaks at low 2θ values, while there are more differences at higher angles. Interestingly, the diffraction pattern collected for the CO_2_-dosed sample does not feature these additional peaks (Figure S6), and it is more consistent with typical diffraction patterns of diamine-appended Mg_2_(dobpdc) with and without CO_2_.^33,34^ The disappearance of peaks after CO_2_ dosing indicates that CO_2_ adsorption gives rise to a more ordered and higher-symmetry space group for the resulting structure. Additionally, the diffraction peaks for the CO_2_-dosed sample appear at 2θ values lower than those for pip2–Mg_2_(dobpdc), indicating an expansion of the crystal lattice to accommodate CO_2_.

Single-crystal and powder X-ray diffraction analysis of other diamine-appended Mg_2_(dobpdc) and Mg_2_(olz) frameworks has revealed that these structures adopt various trigonal space groups,^34,36^ although the diffraction pattern collected for pip2–Mg_2_(dobpdc) could not be indexed to any trigonal space group due to the presence of the additional peaks noted above. Attempts to index the pattern of activated pip2–Mg_2_(dobpdc) with lower symmetry space groups (either monoclinic or triclinic) were unsuccessful, although some extra peaks that could not be indexed with trigonal space groups were indexed by lower symmetry space groups (Figure S13 and Tables S1 and S2). The difficulties encountered with indexing the diffraction pattern to a single space group could be indicative of the presence of two distinct phases of pip2–Mg_2_(dobpdc) due to different orientations of the bulky diamine in the framework pores.

In contrast, the diffraction pattern collected for pip2–Mg_2_(dobpdc) dosed with CO_2_ could be successfully indexed to the space group *P*3_2_21 (Table S2), consistent with the space group assignment for other diamine-appended Mg_2_(dobpdc) and Mg_2_(olz) frameworks dosed with CO_2_.^33,34^ To locate physisorbed CO_2_ in the structure, a calculated structure for pip2–Mg_2_(dobpdc) featuring one CO_2_ per diamine in the form of ammonium carbamate chains was generated using DFT with vdW corrections (see Experimental Section for details).^38^ We then introduced one additional CO_2_ molecule for every two metal sites in the unit cell of the structure to simulate the uptake of half an equivalent of CO_2_ per diamine and performed geometry optimization to allow the CO_2_ molecule to rearrange in the pore. Repeating this calculation with different starting coordinates for CO_2_ yielded three candidate structures that were used for Rietveld refinement of the diffraction pattern for the CO_2_-dosed framework. In the first of these structures, the physisorbed CO_2_ is located near one of two adjacent pip2 moieties in the *ab* plane (structure A, Figure S14). In the second structure, CO_2_ is located in the center of the pore (structure B, Figure S16), and in the third structure, CO_2_ is located in a pocket formed by two carbamates and the linker (structure C, Figure S18).

**Figure 5:**
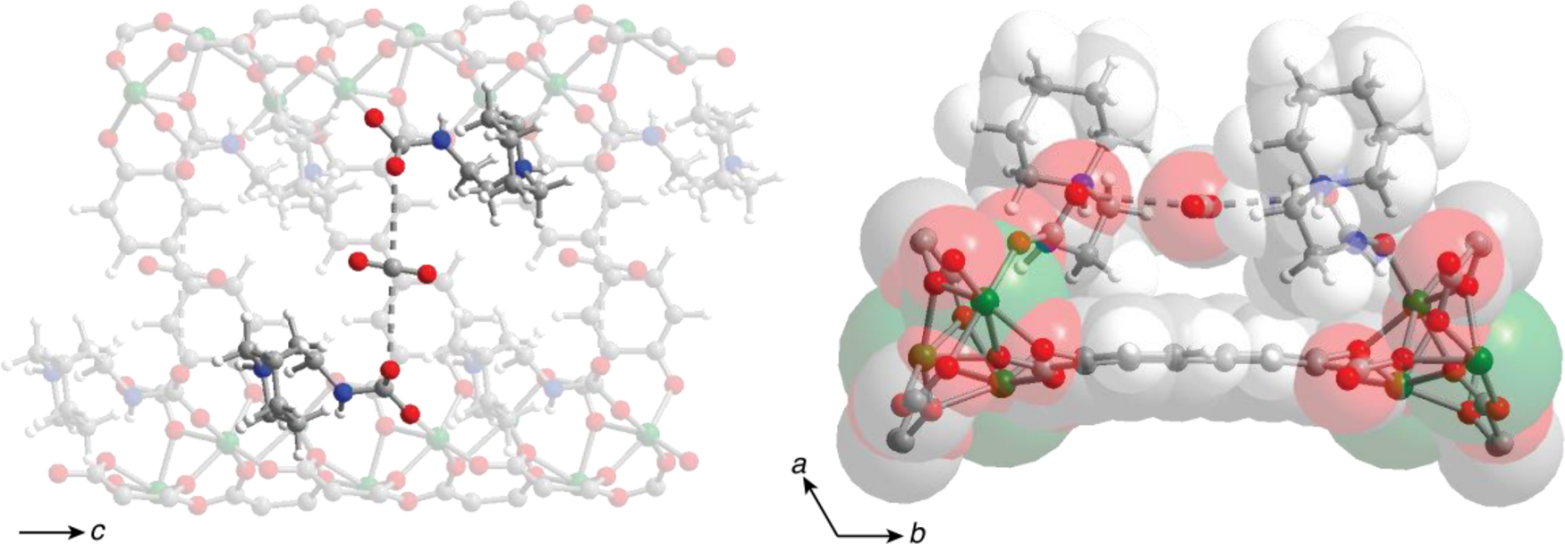
Views of the structure of (CO_2_)_1.5_–pip2–Mg_2_(dobpdc) determined from Rietveld refinement (left) along the *c* axis and (right) in the *ab* plane. Green, red, blue, gray, and white spheres represent Mg, O, N, C, and H, respectively.

When the occupancy of the extra CO_2_ molecule was freely refined in the candidate structures, structures A and B gave physically unreasonable negative occupancies, while structure C yielded a positive occupancy for extra CO_2_ after the initial refinement. Further refinement of the atomic coordinates of structure C yielded a final structure for the CO_2_-dosed pip2–Mg_2_(dobpdc) (Figure 5, Figures S20 and S21, and Tables S2 and S3). Moreover, our DFT calculations with vdW corrections show that structure C is more stable than structures A and B by 10.1 and 8.0 kJ/mol, respectively. As shown in Figure 5, the physisorbed CO_2_ molecules run along the framework channels and are located in the region between adjacent ammonium carbamate chains. The distance between the carbon of the CO_2_ molecule and the oxygen atom of each carbamate is 3.4(2) Å (see Table S3). Based on the van der Waals radii of carbon and oxygen (1.70 and 1.52 Å, respectively),^45^ this distance suggests that the physisorbed CO_2_ molecules are engaged in stabilizing interactions with the ammonium carbamate chains. The physisorbed CO_2_ was ultimately refined to an occupancy of 59(2)%; because it is located at a special position (Wyckoff position 3b) of the *P*3_2_21 space group, this occupancy translates to an uptake of approximately 0.3 equiv per carbamate, consistent with the CO_2_ uptake determined from isobaric and isothermal analyses.

To further support our assignment of the position of physisorbed CO_2_, we used vdW-corrected DFT to simulate geometry-optimized structures for pip2–Mg_2_(dobpdc), pip2–Mg_2_(dobpdc) loaded with 0.5 equiv of CO_2_ per diamine, and pip2–Mg_2_(dobpdc) loaded with 1.5 equiv of CO_2_, the latter based on the final structure discussed above. Using these optimized structures, we calculated the CO_2_ binding energies and NMR chemical shifts associated with species formed after loading of 0.5 and 1.5 equiv of CO_2_ per diamine (see Tables S4 and S5). For pip2–Mg_2_(dobpdc) loaded with 1.5 CO_2_ per diamine, the computed binding energy is within 5 kJ/mol of the corresponding experimental Δ*h*_ads_ value (−48.6 versus −53(1) kJ/mol, respectively), and the simulated carbamate ^13^C NMR shift of 165.9 ppm is comparable to the experimental peak position at 162.5 ppm. Taken together, the spectroscopic and computational results clearly support a mechanism of CO_2_ uptake in pip2–Mg_2_(dobpdc) involving initial chemisorption of CO_2_ at half of the diamine sites, followed by a dual chemisorptive/physisorptive process wherein binding of CO_2_ at the remaining diamine sites is accompanied by uptake of an additional half equivalent of physisorbed CO_2_.

### Adsorption Performance under Simulated Landfill Gas

**Figure 6:**
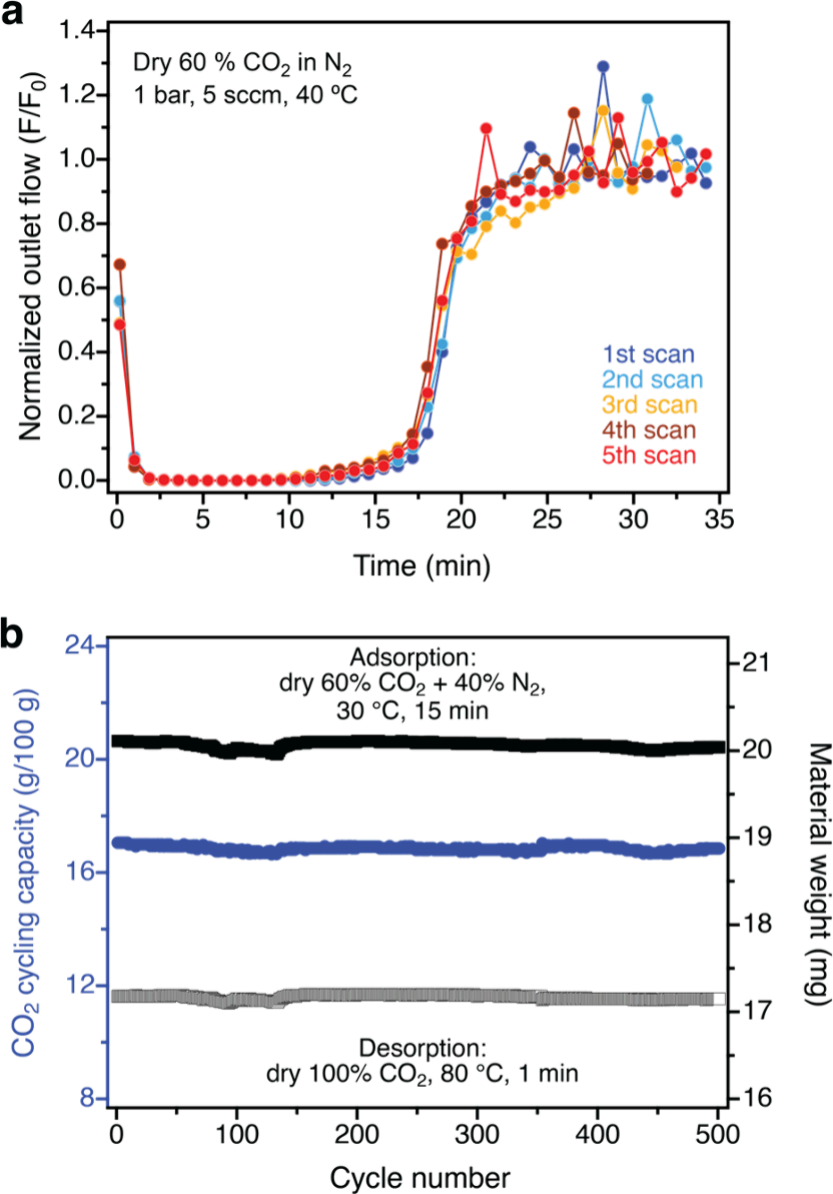
(a) Breakthrough data for pip2–Mg_2_(dobpdc) exposed to flowing dry 60% CO_2_ in N_2_ (∼1 bar, 5 sscm) at 40 °C. The material adsorbed 5.1 ± 0.2 mmol of CO_2_ per gram under these conditions. Note that the nonzero flow detected at the earliest time points is due to residual gas in the tubing downstream from the breakthrough column, resulting from purging the manifold with the gas mixture before the start of each run (see Experimental section for details). This initial flow does not affect the breakthrough capacity calculation. (b) Thermogravimetric temperature–swing cycling data collected for pip2–Mg_2_(dobpdc) at atmospheric pressure; adsorption: 30 °C, 60% CO_2_ in N_2_, 15 min; desorption: 80 °C, CO_2_, 1 min.

As noted above, pip2–Mg_2_(dobpdc) exhibits a high CO_2_ capacity at pressures relevant to the concentration of CO_2_ in landfill gas, which typically contains 40–60% methane, 40–60% CO_2_, and a balance of N_2_ and small amounts of organic compounds.^39−41^ Both methane and CO_2_ are potent greenhouse gases, and methane has a global warming potential nearly 30 times that of CO_2_.^46^ Capturing and refining methane from landfill gases have the potential to significantly reduce greenhouse gas emissions and also provide a valuable energy source. The generation of purified methane from landfill gas involves the removal of moisture and impurities, followed by removal of CO_2_.^46^ To evaluate the potential of pip2–Mg_2_(dobpdc) for this separation, we carried out breakthrough experiments using a custom-built breakthrough apparatus and binder-free pellets of the material (see Experimental Section for details). For safety reasons, a blend of CO_2_ in N_2_ was employed for the experiments instead of the use of CO_2_ and CH_4_. A stream of dry 60% CO_2_ in N_2_ at 25 °C and ambient pressure was passed through the breakthrough column, and the outlet gas composition and flow rate were monitored over time. As shown in Figure 6a, a sharp breakthrough of CO_2_ occurred after 15 min, and the CO_2_ uptake capacity of the material under these conditions was determined to be 5.1 mmol/g. This high capacity remained unchanged over the course of four consecutive desorption (80 °C under flowing He) and breakthrough cycles (Figure 6a), and ^1^H NMR spectroscopy analysis of a digested sample of the framework following this cycling experiment revealed that diamine loading remained at approximately 100%.

The framework also exhibits excellent stability to long-term TGA adsorption/desorption cycling (Figure 6b; adsorption: 15 min, dry 60% CO_2_ in N_2_ at 1 atm (Figure S24); desorption: 1 min, dry CO_2_ at 1 atm and 80 °C). Over the course of 500 cycles, pip2–Mg2(dobpdc) exhibited a stable capacity of ∼16.9 g/100 g (or 3.83 mmol/g; note that this capacity is slightly lower than that determined from the breakthrough experiments as a result of the slightly higher adsorption temperature of 30 °C). Powder X-ray diffraction analysis of the material after the cycling experiment revealed that it remained highly crystalline (Figure S25), and ^1^H NMR spectroscopy analysis of a digested framework sample revealed that diamine loading remained at ∼100%. These results highlight the excellent stability of pip2–Mg_2_(dobpdc) to long-term cycling under conditions relevant to CO_2_ capture from landfill gas.

## Conclusions

We have discovered a new diamine-appended metal–organic framework, pip2–Mg_2_(dobpdc), that captures CO_2_ via an unprecedented mixed chemisorption/physisorption mechanism that endows it with a capacity of nearly 1.5 equiv of CO_2_ per diamine, significantly more than previously reported materials in this class (typically <1.2 equiv per diamine). This behavior is associated with two-step CO_2_ uptake in isothermal and isobaric adsorption data, involving sequential capture of ∼0.5 equiv of CO_2_ per diamine in the first step, followed by ∼1 equiv of CO_2_ per diamine in the second step. *In situ* DRIFTS and solid-state magic angle spinning NMR spectroscopy data support a mechanism in which uptake in the first step is associated with the formation of ammonium carbamate chains at half of the diamine sites in the material, followed by uptake of additional CO_2_ via chemisorption at the remaining diamine sites and physisorption of CO_2_ between resulting adjacent ammonium carbamate chains. Importantly, pip2–Mg_2_(dobpdc) retains its high capacity and exhibits exceptional stability over the course of breakthrough and extended cycling experiments using a simulated landfill gas. Ultimately, these results suggest that, with judicious choice of diamine, it may be possible to design a new class of amine-appended Mg_2_(dobpdc) (and Mg_2_(olz)^36^ materials with significantly enhanced capacities as a result of a mixed chemisorption and physisorption mechanism, as demonstrated here. Additional studies are ongoing to understand the role of the diamine structure and pore environment in facilitating this adsorption mechanism.

## Experimental Section

### General Procedures

All of the experiments are carried out in the air, unless noted otherwise. All reagents and solvents were purchased from Sigma-Aldrich at reagent-grade purity or higher and used without further purification. The ligand H_4_dobpdc was purchased from Hangzhou Trylead Chemical Technology Co. Ultrahigh purity (>99.998%) gases used for all adsorption measurements were purchased from Praxair, as well as the custom gas blends of 60% CO_2_ in N_2_. The solution-phase ^1^H nuclear magnetic resonance (NMR) spectra were collected on a Bruker AMX 400 MHz NMR spectrometer for digested framework samples, which were referenced to residual dimethyl sulfoxide (δ = 2.50 ppm).

### Synthesis of Mg_2_(dobpdc)

The metal–organic framework Mg_2_(dobpdc) was synthesized following the previously reported procedure.^25−32^ Generally, Mg(NO_3_)_2_·6H_2_O (11.6 g, 45.2 mmol) and H_4_(dobpdc) (9.75 g, 35.6 mmol) were dissolved in a 55:45 (v:v) mixture of methanol and *N*,*N*-dimethylformamide (DMF) (total volume of 200 mL) with sonication. The resulting solution was filtered to remove undissolved particles and transferred to a 350 mL high-pressure round-bottom flask equipped with a stir bar. The flask was sealed with a Teflon cap and then heated at 120 °C in an oil bath for 20 h with stirring (300 r.p.m.). After this time, an insoluble white solid had formed. The mixture was allowed to cool to room temperature, and the solid product was filtered using a Buchner funnel. The solid was then soaked in 300 mL of fresh DMF at 80 °C for 6 h and then isolated again via filtration. This process was repeated two more times, and the resulting solid was then soaked in 300 mL of fresh methanol at 60 °C for 6 h and then isolated via filtration; this process was repeated two more times. The resulting solid Mg_2_(dobpdc) was stored in fresh methanol at room temperature. The powder X-ray diffraction pattern obtained for this material (Figure S1) and the calculated Langmuir surface area (77 K, N_2_) of 4000 m^2^/g (Figure S3) are consistent with that previously reported for Mg_2_(dobpdc).^25−32^

### Synthesis of pip2–Mg_2_(dobpdc)

Methanol-solvated Mg_2_(dobpdc) (150 mg) was dispersed in a 5 mL solution of 20% pip2 in toluene. The powder was soaked for 18 h and then filtered and washed with fresh toluene in a Buchner funnel (3 × 20 mL) at room temperature to remove as much residual diamine as possible prior to activation. The diamine-appended MOF was then activated at 130 °C for 1 h under flowing N_2_ in a Schlenk flask equipped with a rubber septum and venting needle (the activation temperature was determined by the thermogravimetric decomposition analysis; see Figure S5). Diamine loading was determined by solution-phase ^1^H NMR spectroscopy analysis of a digested sample of the material. Briefly, ∼2 mg of material was suspended in 0.5 mL of dimethyl sulfoxide-*d*_6_ and 100 μL of DCl solution (35 g/100 g in D_2_O, ≥ 99 atom % D) was added to dissolve the sample.^32^ Surface areas, powder X-ray diffraction patterns, and decomposition profiles are presented in Figures S4–S6.

### Thermogravimetric Analysis

Thermogravimetric analysis (TGA) data were collected by using a TA Instruments Discovery thermogravimetric analyzer. Thermogravimetric decomposition experiments were carried out under 100% N_2_ with a temperature ramping rate of 2 °C/min from 30 to 600 °C with a gas flow rate of 25 mL/min. Masses were not corrected for buoyancy effects. Isobaric data under pure CO_2_ were collected at ambient pressure using a gas flow rate of 25 mL/min. Prior to isobar collection, pip2–Mg_2_(dobpdc) was first activated by heating at 120 °C for 30 min under flowing N_2_. The inlet gas was then switched to 100% CO_2_, and the sample was held isothermally at 120 °C under flowing 100% CO_2_ for 30 min to completely purge the system of N_2_. Adsorption isobar data were obtained while slowly cooling the sample to 30 °C with a ramping rate of 1 °C/min, and desorption data were collected upon then heating the sample to 130 °C at the same ramping rate. The reported two step temperatures from the adsorption isobar were determined using the inflection points of the adsorption steps, and the desorption temperature was determined at the point of closure of the hysteresis loop.

For analysis of adsorption kinetics (see Figure S24), the sample was first activated at 120 °C for 30 min under a 100% dry N_2_ stream to remove remaining unreacted amine in the framework pores and then cooled to 30 °C prior to the kinetics experiments. The inlet gas was then switched to 60% CO_2_ in N_2_ at 1 bar, and the sample was held isothermally for 60 min to study the adsorption kinetics under conditions intended to simulate exposure to landfill gas (Figure S21).

For the cycling experiments, the conditions were based on the adsorption/desorption kinetics results. The materials were first activated at 120 °C for 30 min under a 100% dry N_2_ stream to remove the extra diamine in the framework pores and then ramping to 30 °C prior the cycling experiments. The inlet gas was then switched to dry 60% CO_2_ in N_2_ (∼1 bar) at 30 °C, and the sample was held isothermally for 15 min to adsorb CO_2_; the furnace was then ramped to 80 °C and held isothermally for 1 min as 100% dry CO_2_ (∼1 bar) was flowed over the sample to desorb CO_2_. A total of 500 adsorption/desorption cycles were performed.

### Breakthrough Measurements

Breakthrough experiments were conducted using pip2–Mg_2_(dobpdc) to gauge the separation performance of the material under a multicomponent dynamic stream intended to simulate landfill gas. Experiments were carried out using a custom-built breakthrough apparatus consisting of Swagelok fittings and 1/8″ copper tubing connecting gas flow to the sample holder or bypassing the sample holder. Cylinders of CO_2_ and N_2_ were connected to the breakthrough manifold by using Alicat mass flow controllers. Gas flow was controlled to achieve 60% CO_2_ and 40% N_2_ gas streams with a flow rate of 5 sccm. In particular, prior to flowing the mixture through the pip2–Mg_2_(dobpdc) sample, the gas mixture was equilibrated by flowing through the breakthrough manifold with the sample column closed off. The outlet flow rate was verified using an Agilent ADM Flow Meter, and the outlet gas stream composition was verified using an SRI Instruments 8610 C GC equipped with a 6′ Haysep D column maintained at 110 °C. This purging of the manifold leads to the initial nonzero flow rate in the first few minutes of the breakthrough data in Figure 6.

The pip2–Mg_2_(dobpdc) sample was pelletized using a pellet die with 1 in. diameter and separated with a 20–40 mesh sieve. A sample of pellets (460 mg) was loaded into the sample holder and activated at 80 °C and under flowing He at 5 sccm for 20 min. Then, the sample was cooled to 25 °C for the breakthrough experiments. The outflow composition and flow rate throughout the breakthrough experiment were analyzed using the same flowmeter and GC specified above. Once CO_2_ had broken through the packed pip2–Mg_2_(dobpdc) bed, the stream was switched to He gas at a flow rate of 5 sccm, and the sample holder was heated to 80 °C to fully desorb CO_2_ from the column prior to subsequent breakthrough measurements.

### Gas Adsorption Isotherms

Carbon dioxide adsorption isotherms were collected using a Micromeritics 3Flex gas adsorption analyzer, and 77 K N_2_ adsorption isotherms were collected on a Micromeritics ASAP 2420 instrument. All gases were 99.998% pure or higher. The temperature was controlled by an oil bath during CO_2_ adsorption isotherm collection and controlled by liquid nitrogen when collecting N_2_ adsorption isotherms. Approximately 50 mg of activated pip2–Mg_2_(dobpdc) powder was transferred to a glass adsorption tube with a Micromeritics Transeal. Samples were regenerated at 100 °C under dynamic vacuum (<10 μbar) for overnight between isotherms. The isotherm data points were considered equilibrated if the pressure change is <0.01% after 11 consecutive equilibration time intervals (15 s).

### Calculation of Differential Enthalpies and Entropies for Adsorption

The differential enthalpy (Δ*h*_ads_) and entropy (Δ*s*_ads_) of CO_2_ adsorption for pip2–Mg_2_(dobpdc) were calculated using the Clausius–Clapeyron equation:
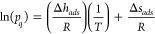


From the isotherm fits, the exact pressure (*p*_*q*_) corresponding to CO_2_ loading (*q*) was determined at different temperatures (*T*) by plotting ln(*p*_*q*_) versus 1/*T* at constant values of *q*. The *y* intercepts of these linear trendlines are equal to – Δ*s*_ads_/*R* at each loading, and the slopes are equal to – Δ*h*_ads_/*R*. Further details are shown in Figure S8–S10.

### Differential Scanning Calorimetry

Differential scanning calorimetry (DSC) experiments were carried out using a TA Instruments Q1000/RCS90 DSC instrument. A sample of activated pip2–Mg_2_(dobpdc) was characterized under an ambient pressure of N_2_ using a gas flow rate of 50 mL/min. The temperature was first ramped to 120 °C and held for 30 min to reactivate the material. Then, the material was cooled to 0 °C and heated to 120 °C (ramp rate of 2 °C/min). DSC data were collected between 25 and 80 °C (Figure S11).

### Solid-State NMR Spectroscopy

All solid-state NMR spectra were collected at 11.7 T using a 4.0 mm Bruker MAS probe with a MAS rate of 10 kHz, except for the ^13^C{^1^H} 2D HETCOR spectrum, which was acquired at 16.5 T using a 3.2 mm Bruker MAS probe with a magic-angle spinning rate of 15 kHz. For data collection, activated pip2–Mg_2_(dobpdc) was packed into a 4.0 mm zirconia NMR rotor inside an argon-filled glovebox before being transferred with a rotor cap (to prevent air exposure) to a home-built gas manifold. For the ^13^CO_2_ dosing experiment, the rotor was transferred to a home-built gas manifold and degassed for 15 min. Subsequently, variable pressures (100, 200, 300, and 1000 mbar) of ^13^CO_2_ gas (Sigma-Aldrich, 99 atom % ^13^C, <3 atom % ^18^O) were dosed into the sample at room temperature and allowed to equilibrate over the course of at least 12 h before capping the rotor for data collection. In particular, after the material was initially dosed at each pressure, the pressure was monitored over time and additional ^13^CO_2_ was dosed into the system until the final pressure was the same as the desired dosing pressure (see our previous study for details on the gas manifold, which allows samples to be closed inside rotors at controlled CO_2_ pressures).^38^

Solid-state ^13^C{^1^H} and ^15^N{^1^H} cross-polarization (CP) NMR spectra were acquired by using an optimal contact time in the range of 1–5 ms with proton Spinal64 decoupling at a B1 field of 57.1 kHz during acquisition. Solid-state MAS ^13^C NMR spectra (11.7 T) for a sample of pip2–Mg_2_(dobpdc) dosed at different ^13^CO_2_ pressures were acquired by a direct pulse with recycle delays (∼100–120 s) and with two-pulse phase modulation ^1^H decoupling at 32 kHz. The quantitative ^13^C NMR spectrum acquired after dosing pip2–Mg_2_(dobpdc) with 1 bar of ^13^CO_2_ was acquired by applying a long recycle delay (1000 s) with continuous-wave ^1^H decoupling (MAS rate of 15 kHz). The ^13^C{^1^H} 2D HETCOR experiments also employed magnetization transfer by cross-polarization with a short contact time of 100 μs, used to selectively show short-range correlations. The ^1^H, ^15^N, and ^13^C chemical shifts were referenced to 1.85 ppm (adamantane), 33.4 ppm (glycine), and 38.48 ppm (adamantane, tertiary carbon—left-hand resonance), respectively.

### *In Situ* Diffuse Reflectance Infrared Fourier Transform Spectroscopy (DRIFTS)

*In situ* DRIFTS data were collected using a Bruker Vertex 70 spectrometer equipped with a glowbar source, KBr beamsplitter, and liquid nitrogen cooled mercury–cadmium–telluride detector. A custom-built diffuse reflectance system with an IR-accessible gas dosing cell was used for all of the measurements. The cell is equipped with a heater controlled by a thermocouple in direct contact with the sample, and the sample atmosphere was controlled by a Micromeritics ASAP 2020Plus gas adsorption analyzer. In a typical experiment, a sample of the activated framework was dispersed in diamond powder (10 wt %) and evacuated at 120 °C before dosing. Known pressures of CO_2_ (99.998%) or ^13^CO_2_ (Sigma-Aldrich, 99 atom % ^13^C, <3 atom % ^18^O) were dosed into the sample using a Micromeritics ASAP 2020Plus gas sorption analyzer. Spectra at 4 cm^–1^ resolution were generated from 128 scans collected over the course of approximately 35 s and collected at 1 min intervals until no further changes were observed. All spectra were processed in pseudoabsorbance units. Difference DRIFTS spectra were generated by subtracting the spectrum of the activated framework from the framework under various dosing conditions, and isotopic difference DRIFTS spectra were generated by subtracting the spectrum of the framework under ^13^CO_2_ from the spectrum of the framework under the same pressure of natural abundance CO_2_.

### Powder X-ray Diffraction Data

Laboratory powder X-ray diffraction data were collected on a Bruker AXS D8 Advance diffractometer with Cu Kα radiation (λ = 1.5418 Å) with sample powders placed on an open-air sample holder. Synchrotron powder X-ray diffraction data were collected at Beamline17-BM-B at the Advanced Photon Source at Argonne National Laboratory using an average wavelength of λ = 0.45399 Å. Activated samples were packed in borosilicate glass capillaries (1.0 mm in diameter) under an N_2_ atmosphere before being attached to a custom-designed gas-dosing cell equipped with a gas valve.^47^ These cells were then mounted on the goniometer head and connected to a gas-dosing manifold for *in situ* diffraction measurements. The sample temperature was controlled using an Oxford CryoSystems Cryostream800. A diffraction pattern was first collected for the activated sample at room temperature, and then the sample was briefly heated to 120 °C under dynamic vacuum before 1 bar of CO_2_ was dosed to the framework. The sample was then cooled to 298 K under a CO_2_ atmosphere. Diffraction patterns were recorded using a PerkinElmera-Si FlatPanel detector and monitored to confirm that the materials had reached equilibrium under gas-dosing conditions. Diffraction patterns were analyzed using TOPAS–Academic v6.1.^48^

Unit cell parameters for CO_2_-dosed pip2–Mg_2_(dobpdc) were obtained by structureless Pawley refinement using TOPAS–Academic v6.1.^48^ The backgrounds of the pattern were modeled with Chebyshev polynomial functions. Peak shapes were described with the fundamental parameter approach. Using the parameters obtained by the Pawley refinement, Rietveld refinement was performed with the structural models of CO_2_-dosed pip2–Mg_2_(dobpdc) obtained from DFT calculations, as discussed (see Figures S13, S15, and S17; see DFT Calculations below for details of the geometry optimization).

The occupancy of CO_2_ was first freely refined by using these models, and it was found (as discussed above) that only structure C gave a positive occupancy. For Rietveld refinement of structure C, the positions and atomic displacement parameters of the Mg atom and the atoms of the linker were refined with restraints. The positions and atomic displacement parameters of the C, O, and N atoms of the carbamate group attached to Mg^2+^ were also refined with restraints. For the other atoms of the pip2 moiety, only atomic displacement parameters were refined, while the positions were not refined. The positions were fixed based on the DFT structure, the alkyl chain and the six-membered ring of pip2 are expected to be flexible at the analyzed temperature (298 K), and it may not be reasonable to determine the conformation of pip2 from these data. The position, atomic displacement parameters, and occupancy of physisorbed CO_2_ were refined with restraints on the C–O bond and O–C–O angle, while the central carbon was freely refined at a Wyckoff position 3b between carbamates. The hydrogen atoms were added to the dobpdc^4–^ linker, ammonium carbamate, and pip2 after refinement with a C–H distance of 1.09 Å and a N–H distance of 1.02 Å using the structure edit tool of Mercury. Although the conformation of the ethylene moiety and the six-membered ring of pip2 was not refined owing to their flexibility, the refinement of the Mg_2_(dobpdc) framework and the physisorbed CO_2_ gave a low *R*_wp_ value (6.58%). This analysis supports the idea that the refined structure is a reasonable model for the approximate position of the physisorbed CO_2_. Note, however, that the bond lengths, bond angles, and atomic displacement parameters of the pip2 moiety are not captured in this model.

### DFT Calculations

First-principles density functional theory (DFT) calculations were performed using GBRV pseudopotentials^49^ and the revised Perdew–Burke–Ernzerhof (RPBE)^50^ exchange-correlation functional with the Quantum ESPRESSO plane wave DFT code.^51^ To include the effect of the van der Waals (vdW) dispersive interactions on energetics, we performed structural relaxations with Grimme’s D3 correction for all calculations, as implemented in Quantum ESPRESSO.^52^ Additionally, for all calculations, we used (i) a 1 × 1 × 3 *k*-point grid sampling, (ii) a 60 Ry plane-wave cutoff energy, and (iii) a 600 Ry charge-density cutoff energy. We explicitly treated 10 valence electrons for Mg (2s^2^2p^6^3s^2^), 6 for O (2s^2^2p^4^), 5 for N (2s^2^2p^3^), 4 for C (2s^2^2p^2^), and 1 for H (1s^1^). Using the above input parameters and initial structures obtained from the Rietveld refinement, we fully relaxed both lattice parameters and internal coordinates. The ions were relaxed until the force is less than 1 × 10^–4^ Ry/Bohr.

Isotropic NMR chemical shielding values (δ_iso_) were computed using the linear response method of Yates et al.^53^ implemented in the Vienna ab initio Simulation Package (VASP)^54−57^ according to the equation δ_iso_ = (−δ_iso-DFT_ – δ_ref-DFT_), where δ_ref-DFT_ is a reference value from the ^13^C and ^15^H chemical shifts of adamantane and glycine, respectively, from prior work.^38^

To compute CO_2_ binding energies, we optimized three different structures: (1) a structure of pip2–Mg_2_(dobpdc) prior to CO_2_ adsorption (*E*_MOF_), (2) a structure of the framework interacting with CO_2_ in the gas phase (*E*_CO_2__) within a 15 Å × 15 Å × 15 Å cubic supercell, and (3) a structure of pip2–Mg_2_(dobpdc) with adsorbed CO_2_ (*E*_CO_2_–MOF_). The binding energy (*E*_B_) was then obtained by using the following equation:
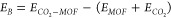


## Supporting Information Available

Additional full experimental characterization data and computational details (PDF)
Supporting simulation and refined structures (ZIP)
