# Supplementary material for: High-Capacity, Cooperative CO2 Capture in a Diamine-Appended Metal–Organic Framework through a Combined Chemisorptive and Physisorptive Mechanism
Source: J Am Chem Soc. 2024 Feb 24;146(9):6072–83. doi: 10.1021/jacs.3c13381 (PMC10921408; doi:10.1021/jacs.3c13381)
Supplement: Supplementary file 1 — ja3c13381_si_001.pdf [file ja3c13381_si_001.pdf]

Supporting Information:

High-Capacity, Cooperative CO_2 Capture in a Diamine-Appended Metal–Organic Framework through a Combined Chemisorptive and Physisorptive Mechanism

Ziting Zhu[1,2,6+], Hsinhan Tsai[1,3,6+], Surya T. Parker[4,6], Jung-Hoon Lee[5,#], Yuto Yabuuchi[1,3,6], Henry Z. H. Jiang[1,3], Yang Wang[1,4], Shuoyan Xiong[1,3], Alexander C. Forse[4,¶], Bhavish Dinakar[4], Adrian Huang[1,3,6], Chaochao Dun[7], Phillip J. Milner[3,†], Alex Smith[5], Pedro Guimarães Martins[4,6], Katie R. Meihaus[1,3], Jeffrey J. Urban[7], Jeffrey A. Reimer[1,4,6], Jeffrey B. Neaton[1,5,6], Jeffrey R. Long[1,3,4,6]*

[1]Institute for Decarbonization Materials, [2]Department of Materials Science and Engineering, [3]Department of Chemistry, [4]Department of Chemical and Biomolecular Engineering, [5]Department of Physics, University of California, Berkeley, California 94720, United States

[6]Materials Sciences Division, [7]Molecular Foundry, Lawrence Berkeley National Laboratory, Berkeley, California 94720, United States

Present address:

[⊥]Computational Science Research Center, Korea Institute of Science and Technology (KIST), Seoul 02792, Republic of Korea

[¶]Department of Chemistry, University of Cambridge, Cambridge, CB2 1EW, United Kingdom

[†]Department of Chemistry and Chemical Biology, Cornell University, Ithaca, New York 14853, United States

*Correspondence to Jeffrey R. Long: jrlong@berkeley.edu

Table of Contents

1. **Mg_2(dobpdc) Synthesis and Characterization** S3
   Powder X–ray Diffraction Data S3
   Thermogravimetric Decomposition Data S3
   N_2 Adsorption Isotherms and Surface Area Calculations S4
2. **Characterization of pip2–Mg_2(dobpdc)** S5
   Thermogravimetric Decomposition Data S5
   N_2 Adsorption Isotherms and Surface Area Calculations S5
   Powder X–ray Diffraction Data Before and After CO_2 Dosing S6
   CH_4 and N_2 Adsorption and Isotherms S7
   CO_2 Differential Enthalpies and Entropies S8
   Calculation of the Approximate Regeneration Energy of pip2–Mg_2(dobpdc) S10
   Solid State NMR of 1 bar $^{13}CO_2$ dosed pip2–Mg_2(dobpdc) S11
   Crystallographic Data S12
   Vdw-DFT Calculation Details S20
   CO_2 Adsorption Kinetics S21
   Powder X–ray Diffraction Data Before and After Cycling S21

1. Mg_2(dobpdc) Synthesis and Characterization

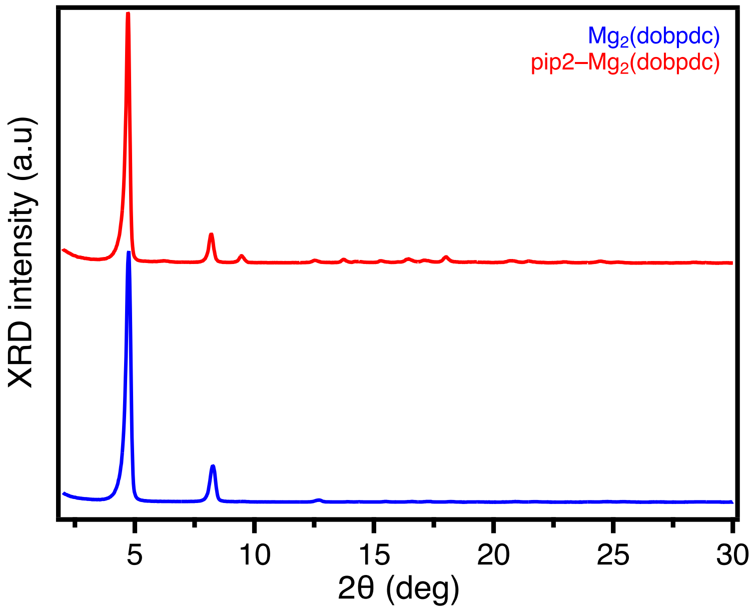

Figure S1. Laboratory powder x–ray diffraction pattern of Mg_2(dobpdc) and pip2–Mg_2(dobpdc) (Cu Kα radiation, λ = 1.5418 Å).

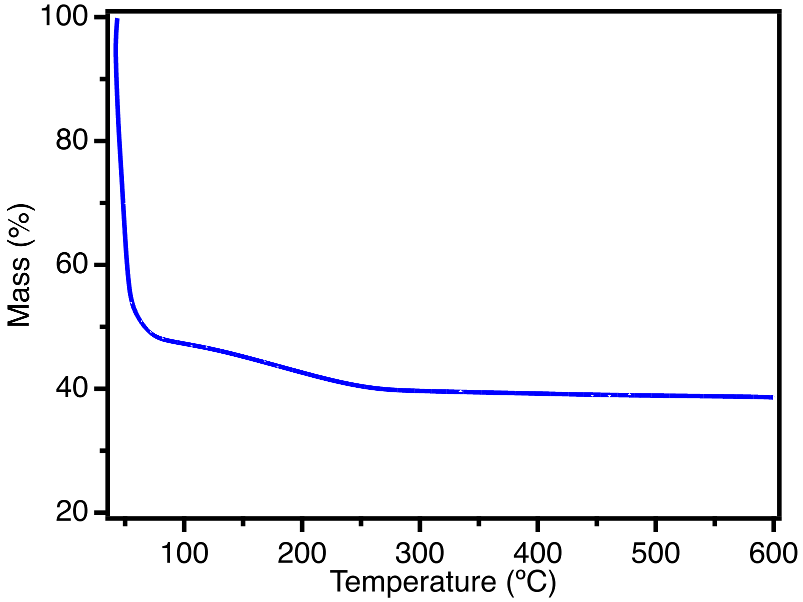

Figure S2. Dry N_2 decomposition profiles of methanol-solvated Mg_2(dobpdc) from 30 to 600 °C. A ramp rate of 2 °C/min was used.

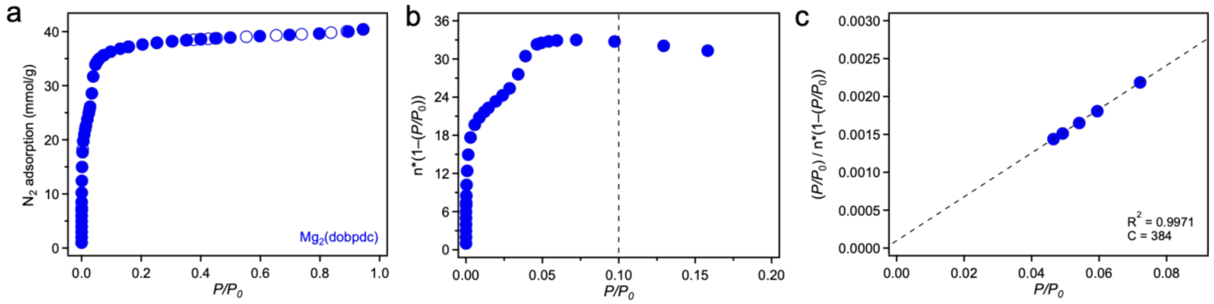

Figure S3. (a) 77 K N_2 adsorption (filled circles) and desorption (open circles) isotherms for activated Mg_2(dobpdc). (b) n*(1-P/P_0) vs. (P/P_0) for Mg_2(dobpdc) isotherm. Only the range blow P/P_0 = 0.10 satisfies the first consistency criterion for applying the BET theory. (c) Plot of the linear region for the BET equation. The calculated BET and Langmuir surface areas are 3330 and 3810 m^2/g, respectively.

2. Diamine–Mg_2(dobpdc) Characterization

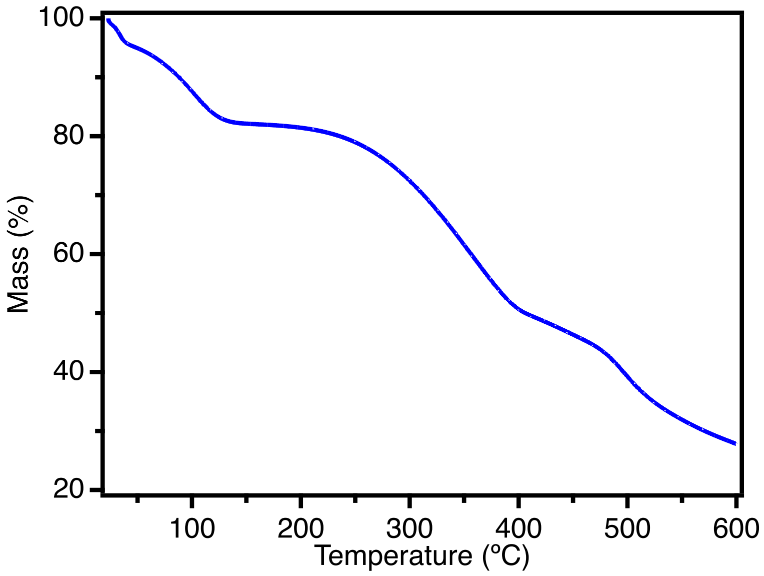

Figure S4. Dry N_2 decomposition profiles of pip2–Mg_2(dobpdc) from 25 °C to 600 °C. A ramp rate of 2 °C/min was used.

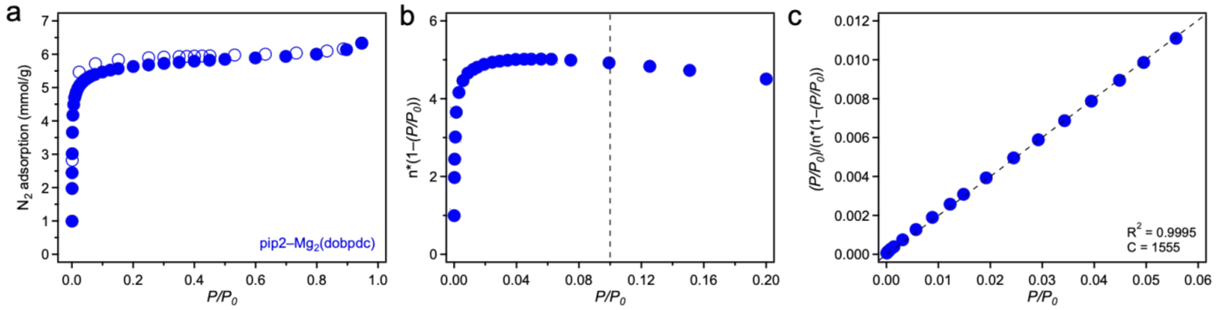

Figure S5. (a) 77 K N_2 adsorption (solid circles) and desorption (open circles) isotherms for activated pip2–Mg_2(dobpdc). (b) n*(1-P/P_0) vs. (P/P_0) for pip2–Mg_2(dobpdc) isotherm. Only the range blow P/P_0 = 0.10 satisfies the first consistency criterion for applying the BET theory. (c) Plot of the linear region for the BET equation. The calculated BET and Langmuir surface areas are 490 and 570 m^2/g, respectively.

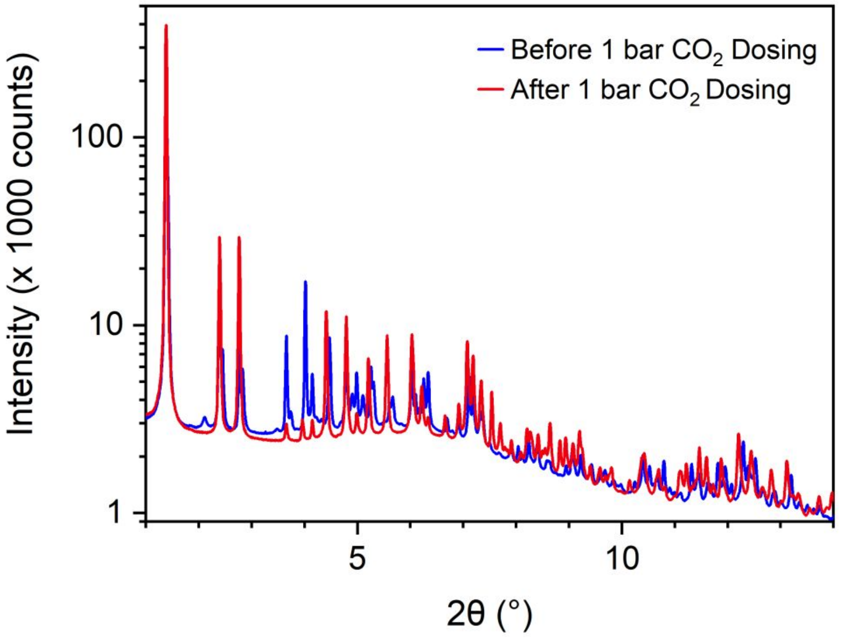

Figure S6. High-resolution powder x-ray diffraction patterns for pip2–Mg_2(dobpdc) before and after dosing with 1 bar CO_2 (λ = 0.45399 Å). The *y*-axis is plotted on a logarithmic scale in order to make small peaks more noticeable.

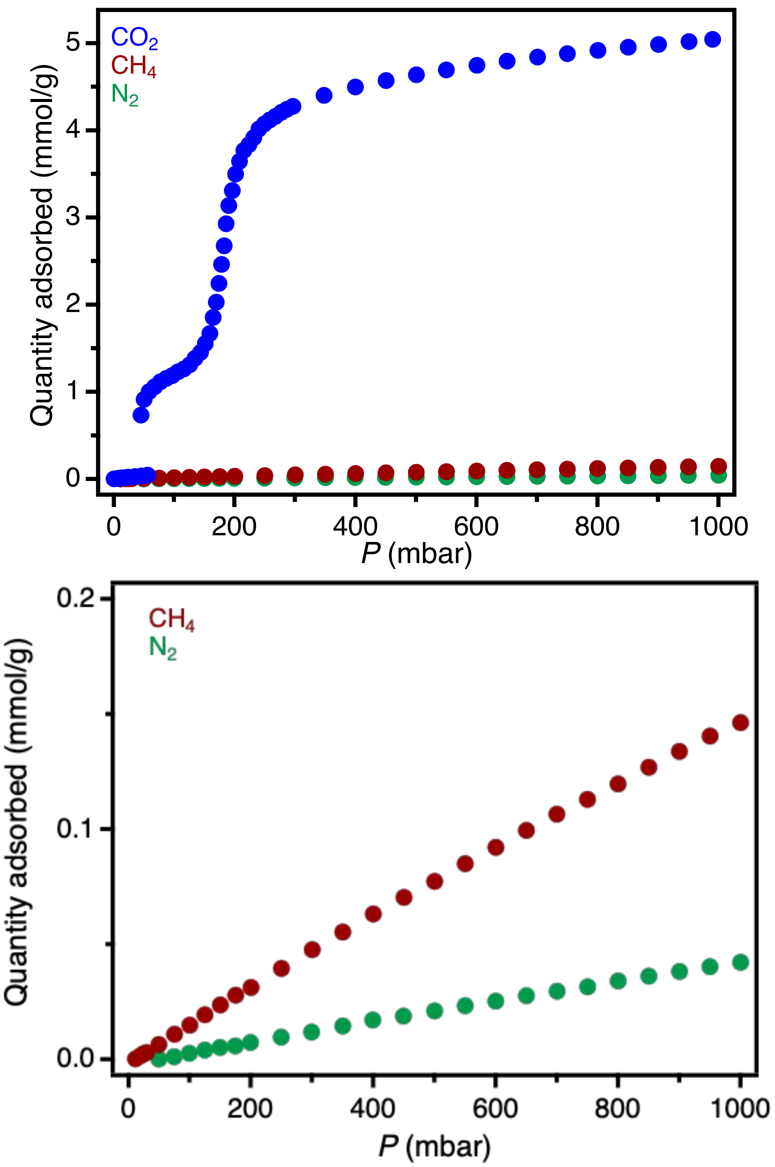

Figure S7. (Upper) Comparison of CO_2, CH_4, and N_2 adsorption isotherms collected for pip2–Mg_2(dobpdc) at 298 K. (Lower) Expanded view of the CH_4 and N_2 for activated pip2–Mg_2(dobpdc) at 298 K.

CO_2 Differential Enthalpies and Entropies of pip2–Mg_2(dobpdc)

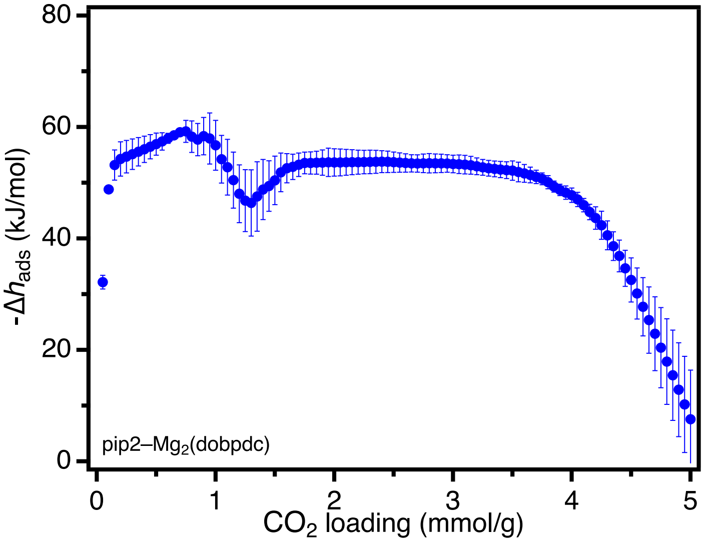

Figure S8. CO_2 differential heats of adsorption for pip2–Mg_2(dobpdc), determined using the Clausius–Clapeyron equation.

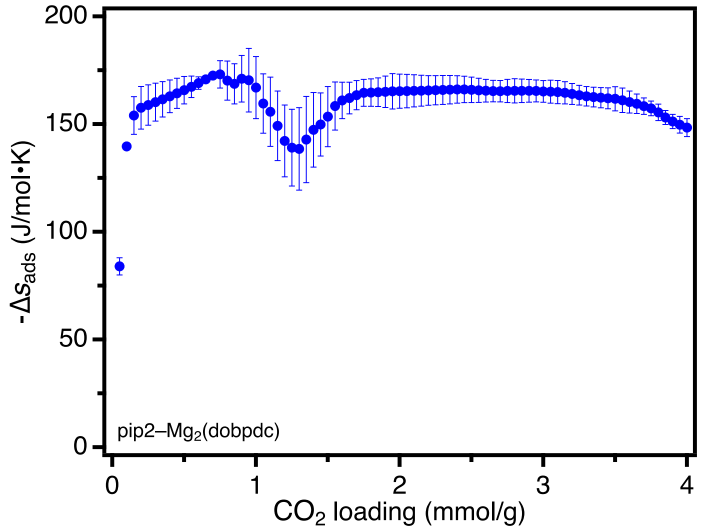

Figure S9. CO_2 differential entropies of adsorption for pip2–Mg_2(dobpdc), determined using the Clausius–Clapeyron equation.

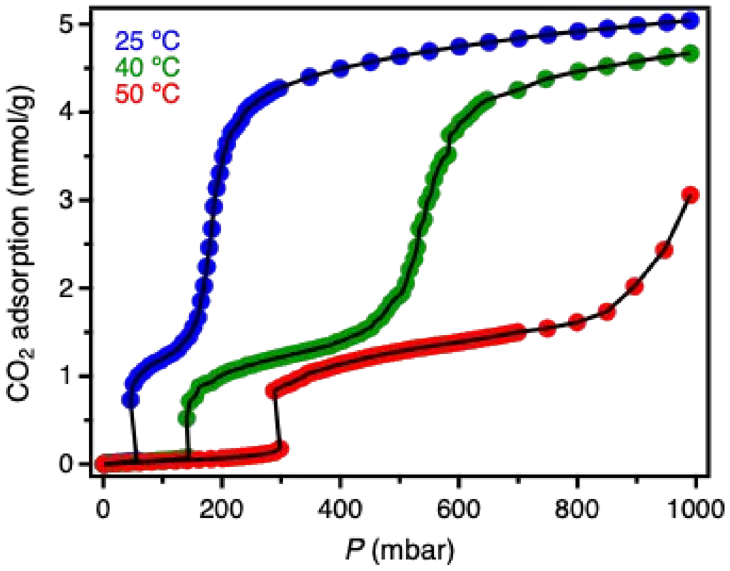

Figure S10. CO_2 adsorption isotherms at 25 °C, 40 °C, and 50 °C for pip2–Mg_2(olz). Isotherms were fitted by linear interpolation (black lines).

Calculation of the Approximate Regeneration Energy of pip2–Mg_2(dobpdc)

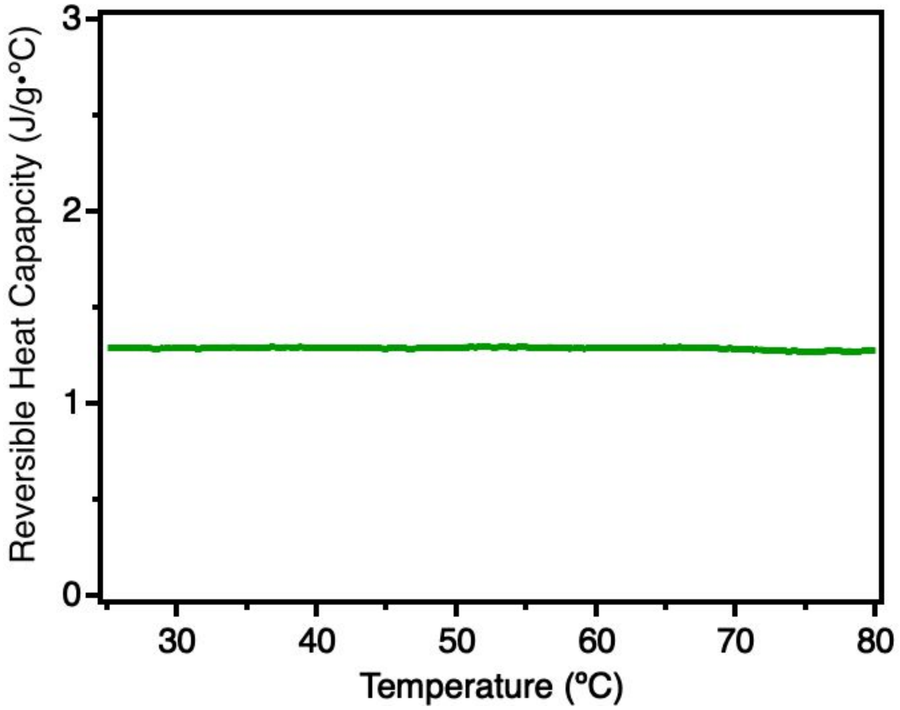

Figure S11. Reversible heat capacity of pip2–Mg_2(dobpdc), determined by modulated differential scanning calorimetry (DSC) under an atmosphere of He. The ramp rate was 2 °C/min. A modulation frequency of 0.75 °C/80 s was used.

Regeneration energies were calculated using the following approach:

$$\frac{MJ}{kg\ CO_2} = \frac{1}{q_{wc}} * \frac{22.72\ mol\ CO_2}{kg\ CO_2} * \frac{1\ MJ}{1000\ kJ}(C_p\Delta T + |\Delta h_{ads}|q_{wc})$$

C_p is the heat capacity in J/g_{MOF}·°C = kJ/kg_{MOF}·°C; C_p of pip2–Mg_2(olz) is 1.29 kJ/kg·°C.

ΔT is the temperature swing; ΔT of pip2–Mg_2(olz) is 55 °C.

Δh_{ads} is the differential enthalpy of adsorption in kJ/mol_{CO2}; Δh_{ads} of pip2–Mg_2(dobpdc) is −59.2±2.0 and −52.5±1.4 kJ/mol for the first and second step, respectively.

q_{wc} is the working capacity in mol_{CO2}/kg_{MOF}; values of q_{wc} for pip2–Mg_2(olz) associated with the first and second step, respectively, are 1.5 and 3.1 mol_{CO2}/kg_{MOF}.

The $C_p\Delta T$ term accounts for the sensible heat, while the $|\Delta h_{ads}|q_{wc}$ term accounts for the regeneration heat.

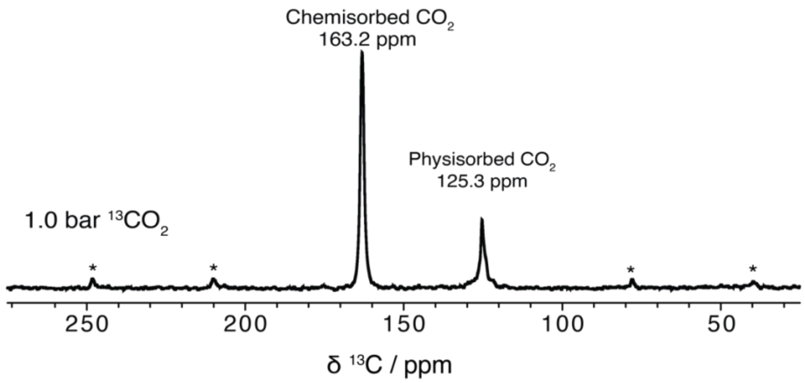

Figure S12. Solid-state MAS ^{13}C (16.4 T) NMR spectrum acquired by direct excitation and with a recycle delay of 1000 s which was sufficiently long to yield quantitative intensities. The pip2–Mg_2(dobpdc) sample was dosed with 1 bar $^{13}CO_2$ pressure for 10 min. A MAS rate of 15 kHz was used with spinning side bands marked by asterisks.

Crystallographic Data

Table S1. Attempt of Pawley refinement of pip2-Mg_2(dobpdc) under vacuum

λ(Å)		0.45399	
Temperature		298 K	
Space Group	$P3_221$	$P2$	$P1$
a (Å)	21.7385(11)	21.698(3)	21.751(3)
b (Å)	21.7385(11)	6.888(5)	6.93(2)
c (Å)	6.8998(9)	20.772(10)	20.8643(10)
α (°)	90	90	89.9(1)
β (°)	90	120.08(1)	119.90(10)
γ (°)	120	90	89.6(1)
Vol. (Å^3)	2823.7(5)	2687(3)	2724(8)
R_{wp} (%)	10.8	11.8	11.8
R_{exp} (%)	1.63	1.63	1.63
R_p (%)	6.81	6.01	5.85
R_{Bragg} (%)	1.82	0.33	0.29
GoF	6.65	7.27	7.28

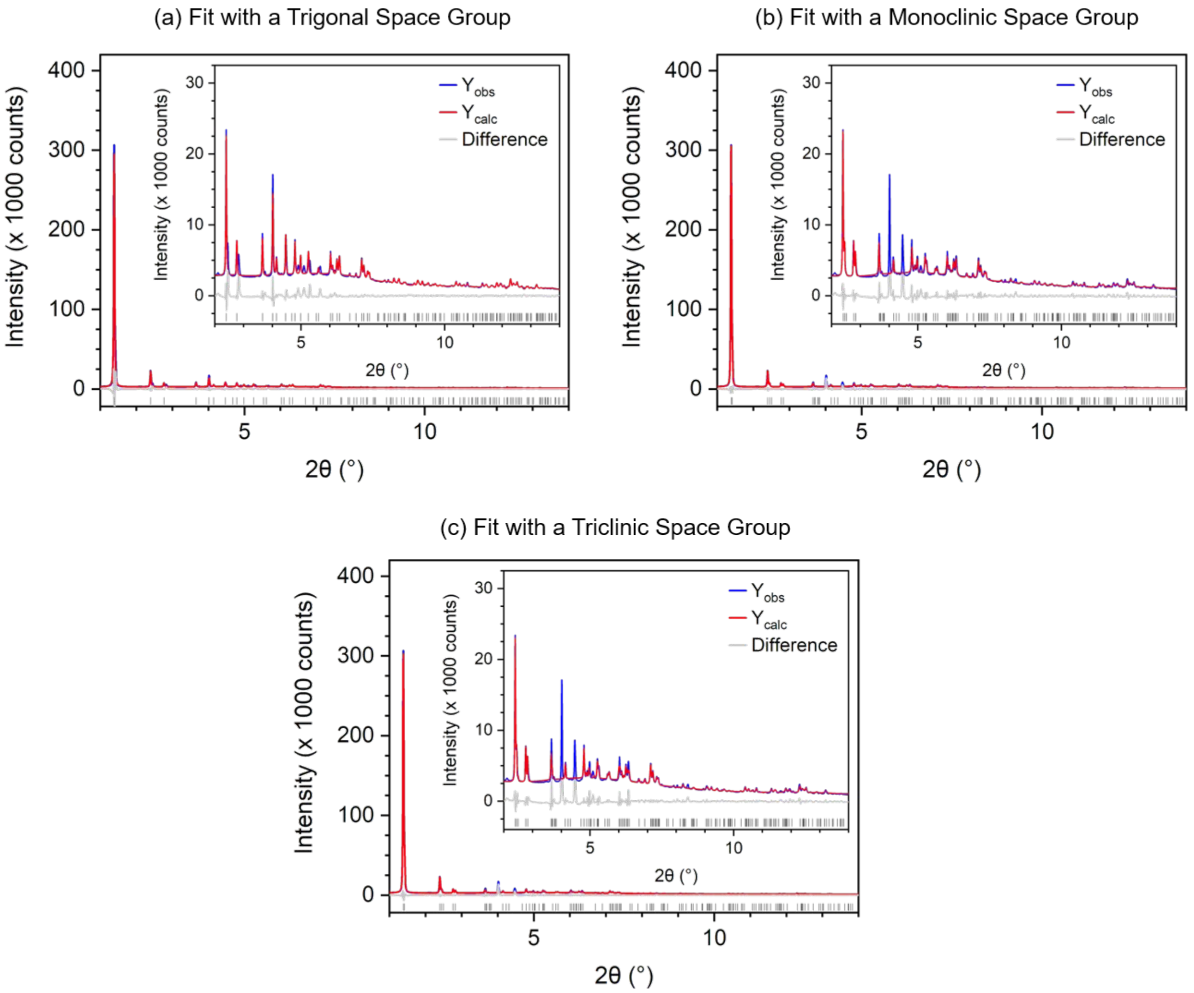

Figure S13. Three attempts of Pawley fit with synchrotron powder x-ray diffraction data collected for pip2–Mg_2(dobpdc) under vacuum at 298 K from 1° to 14°. Blue and red lines represent the observed diffraction patterns and calculated structure patterns, respectively. The gray line represents the difference pattern and the grey tick marks indicate calculated Bragg peak positions for the proposed space group. The inset shows a magnified view of the high angle region. (a) Pawley fit with a trigonal space group $P3_221$, which is a typical space group of amine-appended Mg_2(dobpdc) variants, which does not index several peaks (2θ [°] = 2.44, 2.83, 4.90, 5.10, 5.30, and 5.67). R_{exp} = 1.63%, R_{wp} = 10.8%, R_p = 6.81%, R_{Bragg} = 1.81%. GoF = 6.65. (b) Pawley fit with a monoclinic space group, $P2$. R_{exp} = 1.63%, R_{wp} = 11.8%, R_p = 6.01%, R_{Bragg} = 0.33%. GoF = 7.27. (c) Pawley fit with a triclinic space group, $P1$. R_{exp} = 1.63%, R_{wp} = 11.8%, R_p = 5.85%, R_{Bragg} = 0.29%. GoF = 7.28

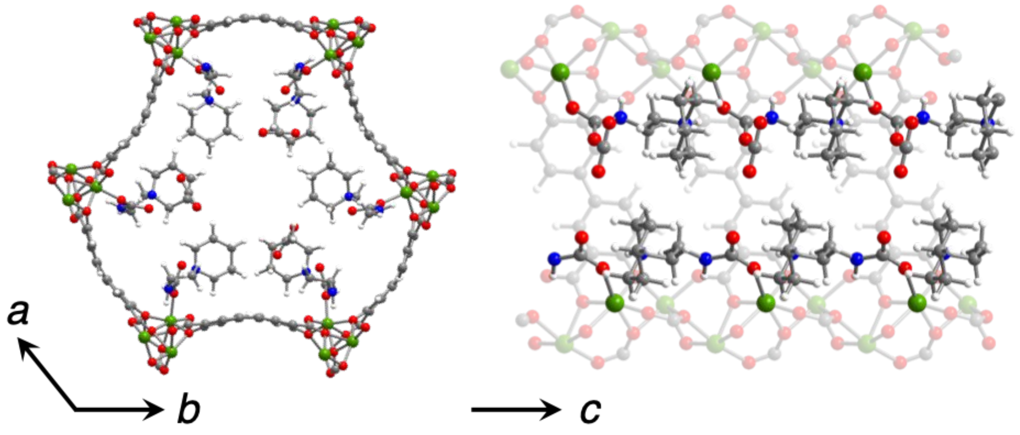

Figure S14. Structure A generated from vdW-corrected DFT for $(CO_2)_{1.5}$–pip2–Mg_2(dobpdc), wherein the physisorbed CO_2 molecules are located near the center of the MOF pore between the pip2 moieties.

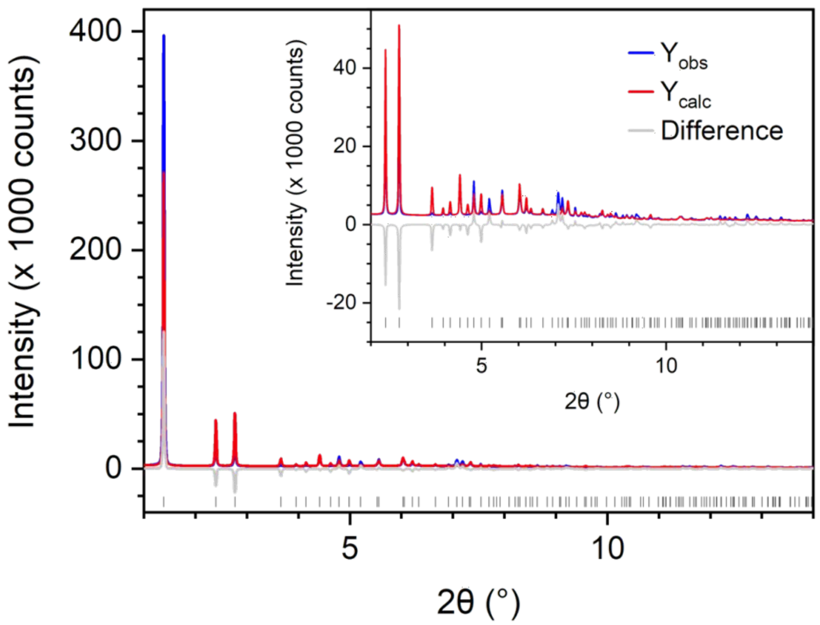

Figure S15. Rietveld refinement with synchrotron powder x-ray diffraction data collected for CO_2-dosed Mg_2(dobpdc) at 298 K from 1.5° to 18.3°. The blue trace represents the experimental diffraction pattern and the red trace represents the calculated pattern based on the DFT structure shown in Figure S15. The gray line represents the difference pattern and the grey tick marks indicate calculated Bragg peak positions. The inset shows a magnified view of the high angle region. Figures-of-merit (as defined by TOPAS): R_{wp} = 28.0%, R_p = 20.2%, R_{exp} = 1.66%, R_{Bragg} = 30.0%, GoF = 16.9. (λ = 0.45399 Å).

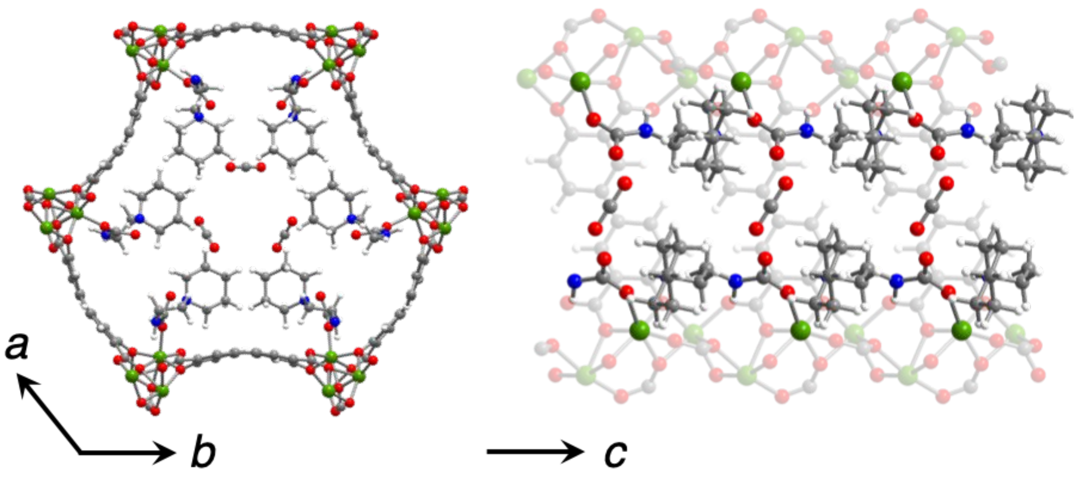

Figure S16. Structure B generated from vdW-corrected DFT for $(CO_2)_{1.5}$–pip2–Mg_2(dobpdc), where the physisorbed CO_2 molecules are located in the center of the pore.

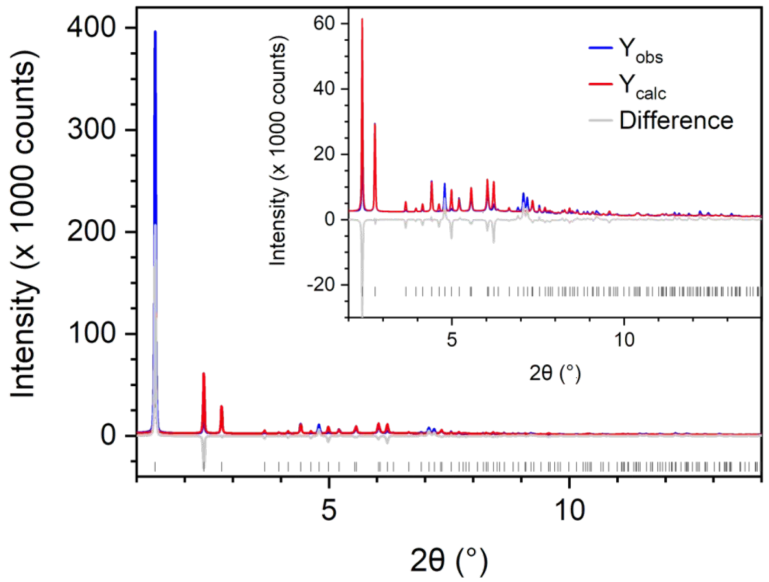

Figure S17. Rietveld refinement with synchrotron powder x-ray diffraction data for CO_2-dosed pip2–Mg_2(dobpdc) at 298 K from 1.5° to 18.3°. The blue trace represents the experimental diffraction pattern, and the red trace represents the pattern calculated for the calculated structure shown in Figure S16 The gray line represents the difference pattern and the grey tick marks indicate calculated Bragg peak positions. The inset shows a magnified view of the high angle region. Figures-of-merit (as defined by TOPAS): R_{wp} = 37.3%, R_p = 26.5%, R_{exp} = 1.66%, R_{Bragg} = 42.9%, GoF = 22.5. (λ = 0.45399 Å).

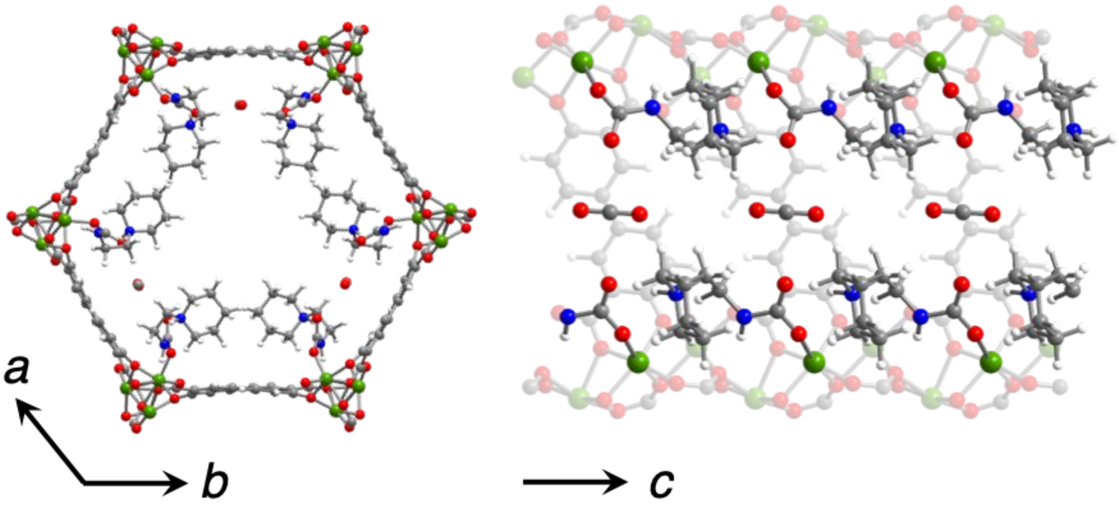

Figure S18. Structure C generated from vdW-corrected DFT for $(CO_2)_{1.5}$–pip2–Mg_2(dobpdc), featuring CO_2 molecules located in a pocket formed by adjacent carbamates in the *ab* plane and the linker.

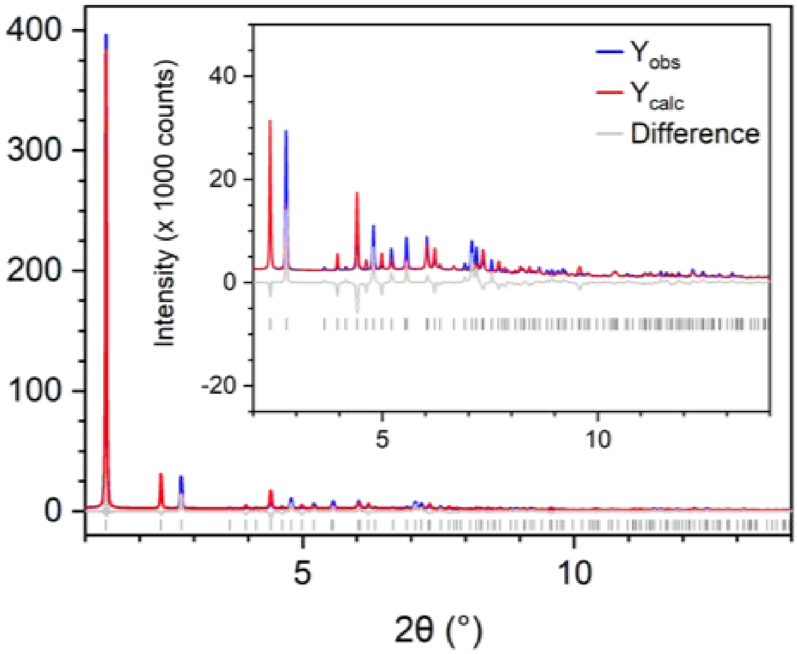

Figure S19. Rietveld refinement of synchrotron powder x-ray diffraction data collected for CO_2-dosed pip2–Mg_2(dobpdc) at 298 K from 1.5° to 18.3°. The blue trace represents the observed diffraction pattern and the red trace corresponds to the calculated pattern based on structure C generated from DFT (Figure S18). The gray line represents the difference pattern and the grey tick marks indicate calculated Bragg peak positions. The inset shows a magnified view of the high angle region. Figures-of-merit (as defined by TOPAS): R_{wp} = 17.4%, R_p = 10.1%, R_{exp} = 1.66%, R_{Bragg} = 8.51%, GoF = 10.5. (λ = 0.45399 Å).

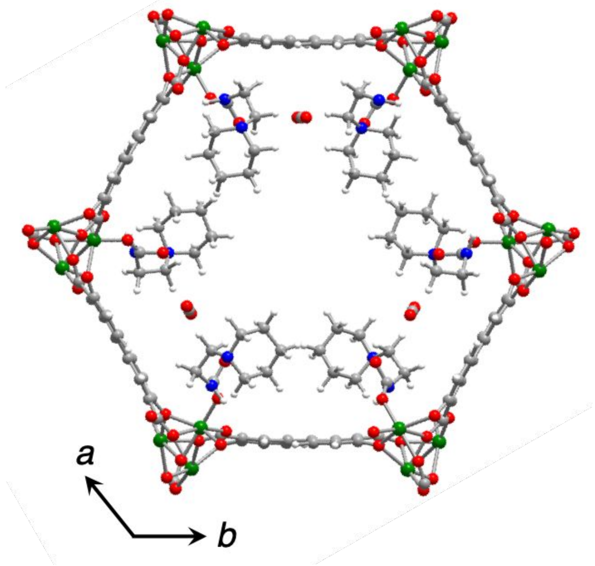

Figure S20. Final structure of $(CO_2)_{1.5}$–pip2–Mg_2(dobpdc) obtained from Rietveld refinement of synchrotron powder x-ray diffraction data for collected after dosing pip2–Mg_2(dobpdc) with 1 bar CO_2 at 298 K from 1° to 14° (λ = 0.45399 Å).

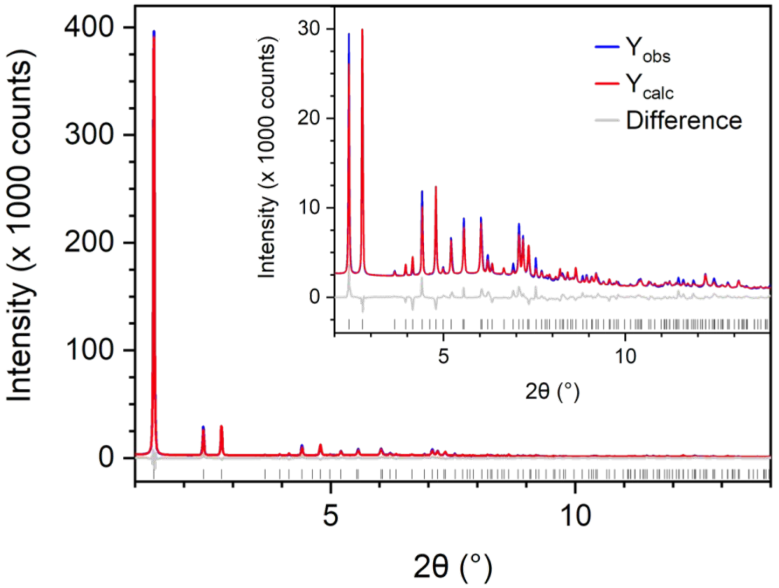

Figure S21. Rietveld refinement with synchrotron powder x-ray diffraction data for $(CO_2)_{1.5}$–pip2–Mg_2(dobpdc) collected under 1 bar CO_2 at 298 K from 1° to 14°. Blue and red lines represent the observed diffraction patterns and calculated structure patterns, respectively. The gray line represents the difference pattern and the grey tick marks indicate calculated Bragg peak positions. The inset shows a magnified view of the high angle region.

Table 2. Refined structure with unit cell and refinement parameters.

	$(CO_2)_{1.5}$–pip2–Mg_2(dobpdc)
λ (Å)	0.45399
Temperature	298 K
Space Group	$P3_221$
a (Å)	21.7346(5)
c (Å)	7.0218(7)
Vol. (Å^3)	2872.7(3)
R_{wp} (%)	6.58
R_{exp} (%)	1.64
R_p (%)	4.40
R_{Bragg} (%)	2.49
GoF	4.02

Table S3. Structural model obtained by Rietveld refinement using a powder x-ray diffraction pattern of pip2–Mg_2(dobpdc) obtained under 1 bar CO_2 at 298 K (see Figure S21). Values in parenthesis indicate one standard deviation from the parameter value. Hydrogens were not refined and are omitted from the model. This Rietveld refinement supports that the refined structure is a reasonable model for the approximate position of the physisorbed CO_2. Space group: $P3_221$. a = 21.7346(5) Å. c = 7.0218(7) Å.

atom	x	y	z	multiplicity	occupancy	U_{iso} (Å^2)*
Mg1	0.599(3)	0.287(5)	0.70(1)	6	1	0.01(1)
O2	0.612(6)	0.228(9)	0.50(3)	6	1	0.05(2)
O3	0.634(5)	0.292(5)	0.12(2)	6	1	0.05(2)
O4	0.57(1)	0.213(8)	−0.10(3)	6	1	0.08(2)
C5	0.59(1)	0.23(1)	0.07(5)	6	1	0.05(2)
C6	0.56(2)	0.17(3)	0.2(1)	6	1	0.175(9)
C7	0.57(2)	0.17(3)	0.4 (1)	6	1	0.175(9)
C8	0.54(2)	0.10(4)	0.51(5)	6	1	0.175(9)
C9	0.51(2)	0.04(3)	0.4(1)	6	1	0.175(9)
C10	0.50(1)	0.03(3)	0.2(1)	6	1	0.175(9)
C11	0.54(3)	0.10(5)	0.11(5)	6	1	0.175(9)
N12	0.48(1)	0.255(8)	0.26(4)	6	1	0.08(1)
C13	0.47(2)	0.30(1)	0.12(6)	6	1	0.149(8)
C14	0.39(2)	0.26(2)	0.04(4)	6	1	0.149(8)
N15	0.38(1)	0.21(1)	0.87(3)	6	1	0.149(8)
C16	0.41(1)	0.16(2)	0.90(4)	6	1	0.149(8)
C17	0.36(2)	0.10(3)	0.03(4)	6	1	0.149(8)
C18	0.056(9)	0.29(1)	0.06(3)	6	1	0.149(8)
C19	0.258(9)	0.11(2)	0.92(4)	6	1	0.149(8)
C20	0.31(2)	0.17(2)	0.80(5)	6	1	0.149(8)
O21	0.501(5)	0.237(7)	0.57(2)	6	1	0.08(1)
O22	0.398(9)	0.217(7)	0.48(3)	6	1	0.08(1)
C23	0.47(1)	0.25(1)	0.45(6)	6	1	0.08(1)
O24	0.01(2)	0.63(1)	0.68(3)	6	0.59(2)	0.02(2)
C25	0	0.67(3)	5/6	3	0.59(2)	0.02(2)

*The thermal parameters were constrained to have the same number for all the atoms in a given moiety (the carboxylate group of the dobpdc^{4-} linker, the oxide group of the dobpdc^{4-} linker, the carbamate group, the two-carbon chain and the six-membered ring of pip2, and the physisorbed CO_2 molecule)

Results from vdW-DFT Calculations

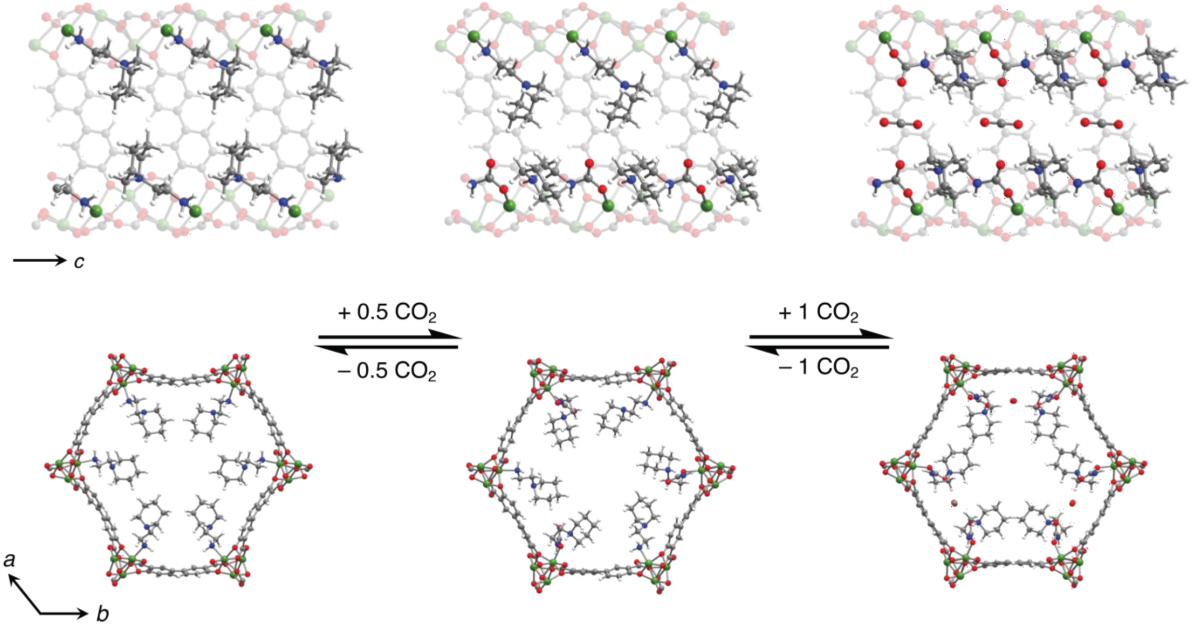

Figure S23. Proposed structures of pip2–Mg_2(dobpdc) (left), pip2–Mg_2(dobpdc) after uptake of 0.5 equiv of CO_2 per diamine (middle), and pip2–Mg_2(dobpdc) after uptake of 1.5 equiv of CO_2 per diamine. Green, red, blue, gray, and white spheres represent Mg, O, N, C, and H, respectively.

Table S4. Computed binding energies (in kJ/mol) for pip2–Mg_2(dobpdc) loaded with 0.5, 1, and 1.5 equiv of CO_2 per diamine.

Loading per diamine	0.5 CO_2	1 CO_2	1.5 CO_2
E_B (kJ/mol)	−72.2	−62.9	−48.6

Table S5. Calculated and Experimental NMR chemical shifts for pip2–Mg_2(dobpdc) loaded with 0.5 and 1.5 equiv of CO_2 per diamine.

	0.5 CO_2/diamine calc (exp)	1.5 CO_2/diamine calc (exp)
δ ^{13}C (ppm)		
COO^-	164.4 (162.5)	165.9 (162.5)
δ ^{15}N (ppm)		
$RNHCOO^-$	82.1 (85.0)	84.4 (85.3)
NHR_3^+	52.9 (51.3)	56.1 (51.5)

CO_2 Adsorption Kinetics Data

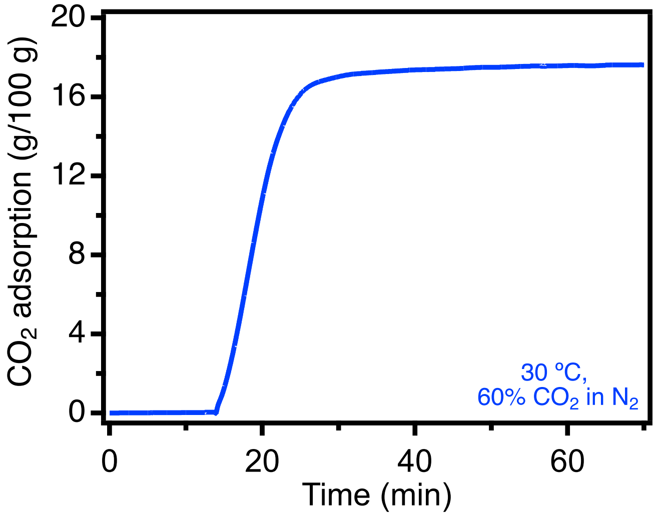

Figure S24. Kinetic trace for CO_2 uptake in pip2–Mg_2(dobpdc) exposed to a stream of 60% CO_2 in N_2 (1 bar) at 30 °C, obtained using a thermogravimetric analyzer. The gas was switched from flowing N_2 (1 bar) to under 60% CO_2 blend with N_2 at 13 min.

Powder x-ray Diffraction Data Before and After Cycling

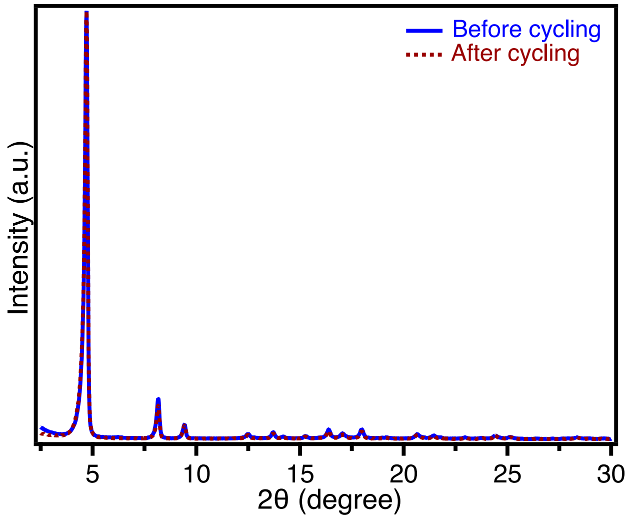

Figure S25. Laboratory powder x-ray diffraction pattern of pip2–Mg_2(dobpdc) material before (solid blue) and after (dash red) cycling test (Cu Kα radiation, λ = 1.5418 Å).

Supplemental Note: Checkcif alerts and responses
start Validation Reply Form
_vrf_PLAT340_Mg2dobpdc_2pip2_3CO2
;
PROBLEM: Low Bond Precision on C-C Bonds .... 0.67692 Ang.
RESPONSE: This amount of precision is due to the nature of the measurement and the framework. This structure is obtained by powder X-ray diffraction. While the precision of this structure might be the lowest among structures refined by powder X-ray diffraction, this framework contains the flexible appended-diamine molecule, whose atomic coordinates are difficult to refine even with hard restraints. The point of this structure is the approximate position of the physorbed CO2, rather than the atomic coordinates of the framework. The low precision of the bondlengths can be overlooked.
;

start Validation Reply Form
_vrf_PLAT414_Mg2dobpdc_2pip2_3CO2
;
PROBLEM: Short Intra D-H..H-X H28 ..H33 . 1.76 Ang.
RESPONSE: Hydrogen atoms were placed after refinement of atomic positions of non-hydrogen atoms, and the positions of hydrogen atoms were not refined. Therefore, a distance between two hydrogen atoms is not meaningful.
;

_vrf_PLAT369_Mg2dobpdc_2pip2_3CO2
;
PROBLEM: Long C(sp2)-C(sp2) Bond C10 - C10_j . 1.70 Ang.
RESPONSE: This amount of precision is due to the nature of the measurement and the framework. This structure is obtained by powder X-ray diffraction. While the precision of this structure might be the lowest among structures refined by powder X-ray diffraction, this framework contains the flexible appended-diamine molecule, whose atomic coordinates are difficult to refine even with hard restraints. The point of this structure is rather the approximate position of the physorbed CO2, rather than the atomic coordinates of the framework. The low precision of the bondlengths can be overlooked.
;
_vrf_PLAT601_Mg2dobpdc_2pip2_3CO2
;
PROBLEM: Unit Cell Contains Solvent Accessible VOIDS of . 443 Ang**3
RESPONSE: This amount of solvent accessible voids is common for highly porous materials, of which this framework is one.
;

_vrf_PLAT351_Mg2dobpdc_2pip2_3CO2

;
PROBLEM: Long C-H (X0.96,N1.08A) C20 - H42_b . 1.18 Ang.
RESPONSE: Hydrogen atoms were placed after refinement of atomic positions of non-hydrogen atoms, and the positions of hydrogen atoms were not refined. Therefore, a distance between the carbon atom and the hydrogen atom is not meaningful.
;

_vrf_PLAT241_Mg2dobpdc_2pip2_3CO2
;
PROBLEM: High 'MainMol' Ueq as Compared to Neighbors of O2 Check
RESPONSE: A single thermal displacement parameter was calculated for all the atoms in the linker as is common practice in the refinement of metal organic frameworks as there is commonly significant disorder among the linker atoms that cannot be refined. In the case of this Mg2(dobpdc) framework, the biphenyl moiety is likely disordered, which contributes to the overall high Uiso for these atoms.
;
_vrf_PLAT242_Mg2dobpdc_2pip2_3CO2
;
PROBLEM: Low 'MainMol' Ueq as Compared to Neighbors of Mg1 Check
RESPONSE: A single thermal displacement parameter was calculated for all the atoms in the linker as is common practice in the refinement of metal organic frameworks as there is commonly significant disorder among the linker atoms that cannot be refined.
;
_vrf_PLAT780_Mg2dobpdc_2pip2_3CO2
;
PROBLEM: Coordinates do not Form a Properly Connected Set Please Do !
RESPONSE: This alert comes from the diamine molecule appended to the Mg site. Because of the flexibility of this moiety, we cannot refine the atomic coordinates of this moiety, and the atomic coordinates are based on a model structure obtained by DFT calculation.
;

_vrf_PLAT353_Mg2dobpdc_2pip2_3CO2
;
PROBLEM: Long N-H (N0.87,N1.01A) N12 - H19 . 1.01 Ang.
RESPONSE: All hydrogen atoms were calculated and therefore the bondlengths are not meaningful.
;
_vrf_PLAT410_Mg2dobpdc_2pip2_3CO2
;
PROBLEM: Short Intra H...H Contact H1 ..H3 . 1.92 Ang.
RESPONSE: All hydrogen atoms were calculated and therefore the bondlengths are not meaningful.

;
_vrf_PLAT411_Mg2dobpdc_2pip2_3CO2
;
PROBLEM: Short Inter H...H Contact H1 ..H2 . 2.13 Ang.
RESPONSE: All hydrogen atoms were calculated and therefore the bondlengths are not meaningful.
;
_vrf_PLAT761_Mg2dobpdc_2pip2_3CO2
;
PROBLEM: CIF Contains no X-H Bonds .... Please Check
RESPONSE: All hydrogen atoms were calculated and therefore the bondlengths are not meaningful.
;

_vrf_PLAT077_Mg2dobpdc_2pip2_3CO2
;
PROBLEM: Unitcell Contains Non-integer Number of Atoms .. Please Check
RESPONSE: The non-integer number of atoms originates from the incomplete occupancy of the physisorbed CO2 molecule. The incomplete occupancy of the physisorbed CO2 agrees with CO2 uptake obtained by CO2 adsorption isobar and isotherm experiments.
;

_vrf_PLAT420_Mg2dobpdc_2pip2_3CO2
;
PROBLEM: D-H Bond Without Acceptor N12 --H28 . Please Check
RESPONSE: Hydrogen atoms were placed after refinement of atomic positions of non-hydrogen atoms, and the positions of hydrogen atoms were not refined. Therefore, a distance between the nitrogen atom and the hydrogen atom is not meaningful.
;
end Validation Reply Form

_vrf_PLAT762_Mg2dobpdc_2pip2_3CO2
;
PROBLEM: CIF Contains no X-Y-H or H-Y-H Angles .... Please Check
RESPONSE: All hydrogen atoms were calculated and therefore the bond angles are not meaningful.
;
