# Supplementary material for: High-Capacity, Cooperative CO2 Capture in a Diamine-Appended Metal–Organic Framework through a Combined Chemisorptive and Physisorptive Mechanism
Source: J Am Chem Soc. 2024 Feb 24;146(9):6072–83. doi: 10.1021/jacs.3c13381 (PMC10921408; doi:10.1021/jacs.3c13381)

checkCIF/PLATON report

You have not supplied any structure factors. As a result the full set of tests cannot be run.

THIS REPORT IS FOR GUIDANCE ONLY. IF USED AS PART OF A REVIEW PROCEDURE FOR PUBLICATION, IT SHOULD NOT REPLACE THE EXPERTISE OF AN EXPERIENCED CRYSTALLOGRAPHIC REFEREE.

No syntax errors found. CIF dictionary Interpreting this report

Datablock: Mg2dobpdc_2pip2_3CO2

```
Bond precision:   C-C = 0.7000 A          Wavelength=0.45415

Cell:              a=21.7441(11)      b=21.7441(11)      c=7.0248(6)
                   alpha=90           beta=90            gamma=120
Temperature:       298 K
```

	Calculated	Reported
Volume	2876.4(4)	2876.4(4)
Space group	P 32 2 1	P 32 2 1
Hall group	P 32 2"	P 32 2"
Moiety formula	C15 H19 Mg N2 O5, 0.445(C O2)	C15 H19 Mg N2 O5, 0.445(C O2)
Sum formula	C15.45 H19 Mg N2 O5.89	C15.45 H19 Mg N2 O5.89
Mr	351.22	351.22
Dx,g cm-3	1.217	1.211
Z	6	6
Mu (mm-1)	0.049	0.000
F000	1108.7	0.0
F000’	1108.74	
h,k,lmax	11,11,3	
Nref	333[195]	
Tmin,Tmax		
Tmin’		

```
Correction method= Not given

Data completeness= 0.00/0.00        Theta(max)=

R(reflections)=                                      wR2(reflections)=
S =                          Npar=
```

The following ALERTS were generated. Each ALERT has the format
test-name_ALERT_alert-type_alert-level.
Click on the hyperlinks for more details of the test.

Alert level A

PLAT340_ALERT_3_A Low Bond Precision on C-C Bonds .... 0.7 Ang.

Author Response: This amount of precision is due to the nature of the measurement and the framework. This structure is obtained by powder X-ray diffraction. While the precision of this structure might be the lowest among structures refined by powder X-ray diffraction, this framework contains the flexible appended-diamine molecule, whose atomic coordinates are difficult to refine even with hard restraints. The point of this structure is the approximate position of the physorbed CO_2, rather than the atomic coordinates of the framework. The low precision of the bondlengths can be overlooked.

PLAT369_ALERT_2_A Long C(sp2)-C(sp2) Bond C10 - C10_j . 1.70 Ang.

Author Response: This amount of precision is due to the nature of the measurement and the framework. This structure is obtained by powder X-ray diffraction. While the precision of this structure might be the lowest among structures refined by powder X-ray diffraction, this framework contains the flexible appended-diamine molecule, whose atomic coordinates are difficult to refine even with hard restraints. The point of this structure is rather the approximate position of the physorbed CO_2, rather than the atomic coordinates of the framework. The low precision of the bondlengths can be overlooked.

PLAT601_ALERT_2_A Unit Cell Contains Solvent Accessible VOIDS of . 443 Ang**3

Author Response: This amount of solvent accessible voids is common for highly porous materials, of which this framework is one.

Alert level B

PLAT241_ALERT_2_B High 'MainMol' Ueq as Compared to Neighbors of O2 Check

Author Response: A single thermal displacement parameter was calculated for all the atoms in the linker as is common practice in the refinement of metal organic frameworks as there is commonly significant disorder among the linker atoms that cannot be refined. In the case of this Mg_2(dobpdc) framework, the biphenyl moiety is likely disordered, which contributes to the overall high Uiso for these atoms.

```
PLAT242_ALERT_2_B Low    'MainMol' Ueq as Compared to Neighbors of        Mg1 Check
```

Author Response: A single thermal displacement parameter was calculated for all the atoms in the linker as is common practice in the refinement of metal organic frameworks as there is commonly significant disorder among the linker atoms that cannot be refined.

```
PLAT369_ALERT_2_B Long   C(sp2)-C(sp2) Bond  C6       - C7       .       1.60 Ang.
```

Author Response: This amount of precision is due to the nature of the measurement and the framework. This structure is obtained by powder X-ray diffraction. While the precision of this structure might be the lowest among structures refined by powder X-ray diffraction, this framework contains the flexible appended-diamine molecule, whose atomic coordinates are difficult to refine even with hard restraints. The point of this structure is rather the approximate position of the physorbed CO2, rather than the atomic coordinates of the framework. The low precision of the bondlengths can be overlooked.

```
PLAT780_ALERT_1_B Coordinates do not Form a Properly Connected Set     Please Do !
```

Author Response: This alert comes from the diamine molecule appended to the Mg site. Because of the flexibility of this moiety, we cannot refine the atomic coordinates of this moiety, and the atomic coordinates are based on a model structure obtained by DFT calculation.

Alert level C

```
PLAT241_ALERT_2_C High   'MainMol' Ueq as Compared to Neighbors of        O21 Check
```

Author Response: A single thermal displacement parameter was calculated for all the atoms in the linker as is common practice in the refinement of metal organic frameworks as there is commonly significant disorder among the linker atoms that cannot be refined. In the case of this Mg2(dobpdc) framework, the biphenyl moiety is likely disordered, which contributes to the overall high Uiso for these atoms.

```
PLAT351_ALERT_3_C Long   C-H (X0.96,N1.08A)  C14      - H6       .       1.11 Ang.
PLAT353_ALERT_3_C Long   N-H (N0.87,N1.01A)  N12      - H19      .       1.01 Ang.
```

Author Response: All hydrogen atoms were calculated and therefore the bondlengths are not meaningful.

```
PLAT410_ALERT_2_C Short Intra H...H Contact  H1       ..H3       .       1.92 Ang.
                                               x-y,-y,1/3-z  =      5_555 Check
```

Author Response: All hydrogen atoms were calculated and therefore the bondlengths are not meaningful.

```
PLAT410_ALERT_2_C Short Intra H...H Contact  H12      ..H12      .       1.94 Ang.
                                               x-y,-y,1/3-z  =      5_555 Check
```

Author Response: All hydrogen atoms were calculated and therefore the bondlengths are not meaningful.

```
PLAT411_ALERT_2_C Short Inter H...H Contact  H1       ..H2       .       2.13 Ang.
                                               x-y,-y,4/3-z  =      5_556 Check
```

Author Response: All hydrogen atoms were calculated and therefore the bondlengths are not meaningful.

```
PLAT761_ALERT_1_C CIF Contains no X-H Bonds ......................     Please Check
```

Author Response: All hydrogen atoms were calculated and therefore the bondlengths are not meaningful.

```
PLAT762_ALERT_1_C CIF Contains no X-Y-H or H-Y-H Angles .........     Please Check
```

Author Response: All hydrogen atoms were calculated and therefore the bond angles are not meaningful.

Alert level G

```
PLAT004_ALERT_5_G Polymeric Structure Found with Maximum Dimension          3 Info
PLAT007_ALERT_5_G Number of Unrefined Donor-H Atoms .............          1 Report
PLAT092_ALERT_4_G Check: Wavelength Given is not Cu,Ga,Mo,Ag,In Ka    0.45415 Ang.
PLAT302_ALERT_4_G Anion/Solvent/Minor-Residue Disorder (Resd  2  )       100% Note
PLAT343_ALERT_2_G Unusual sp?      Angle Range in Main Residue for        C23 Check
PLAT860_ALERT_3_G Number of Least-Squares Restraints ............         56 Note
PLAT981_ALERT_1_G No non-zero f" Anomalous Scattering Values Found     Please Check
PLAT986_ALERT_1_G No non-zero f' Anomalous Scattering Values Found     Please Check
```

---

3 **ALERT level A** = Most likely a serious problem - resolve or explain
4 **ALERT level B** = A potentially serious problem, consider carefully
8 **ALERT level C** = Check. Ensure it is not caused by an omission or oversight
8 **ALERT level G** = General information/check it is not something unexpected

5 ALERT type 1 CIF construction/syntax error, inconsistent or missing data

```
10 ALERT type 2 Indicator that the structure model may be wrong or deficient
 4 ALERT type 3 Indicator that the structure quality may be low
 2 ALERT type 4 Improvement, methodology, query or suggestion
 2 ALERT type 5 Informative message, check
```

It is advisable to attempt to resolve as many as possible of the alerts in all categories. Often the minor alerts point to easily fixed oversights, errors and omissions in your CIF or refinement strategy, so attention to these fine details can be worthwhile. In order to resolve some of the more serious problems it may be necessary to carry out additional measurements or structure refinements. However, the purpose of your study may justify the reported deviations and the more serious of these should normally be commented upon in the discussion or experimental section of a paper or in the "special_details" fields of the CIF. checkCIF was carefully designed to identify outliers and unusual parameters, but every test has its limitations and alerts that are not important in a particular case may appear. Conversely, the absence of alerts does not guarantee there are no aspects of the results needing attention. It is up to the individual to critically assess their own results and, if necessary, seek expert advice.

Publication of your CIF in IUCr journals

A basic structural check has been run on your CIF. These basic checks will be run on all CIFs submitted for publication in IUCr journals (*Acta Crystallographica*, *Journal of Applied Crystallography*, *Journal of Synchrotron Radiation*); however, if you intend to submit to *Acta Crystallographica Section C* or *E* or *IUCrData*, you should make sure that full publication checks are run on the final version of your CIF prior to submission.

Publication of your CIF in other journals

Please refer to the *Notes for Authors* of the relevant journal for any special instructions relating to CIF submission.

Validation response form

Please find below a validation response form (VRF) that can be filled in and pasted into your CIF.

```
# start Validation Reply Form
_vrf_PLAT351_Mg2dobpdc_2pip2_3CO2
;
PROBLEM: Long   C-H (X0.96,N1.08A)  C14      - H6       .       1.11 Ang.
RESPONSE: ...
;
# end Validation Reply Form
```

PLATON version of 28/11/2022; check.def file version of 28/11/2022

Datablock Mg2dobpdc_2pip2_3CO2 - ellipsoid plot

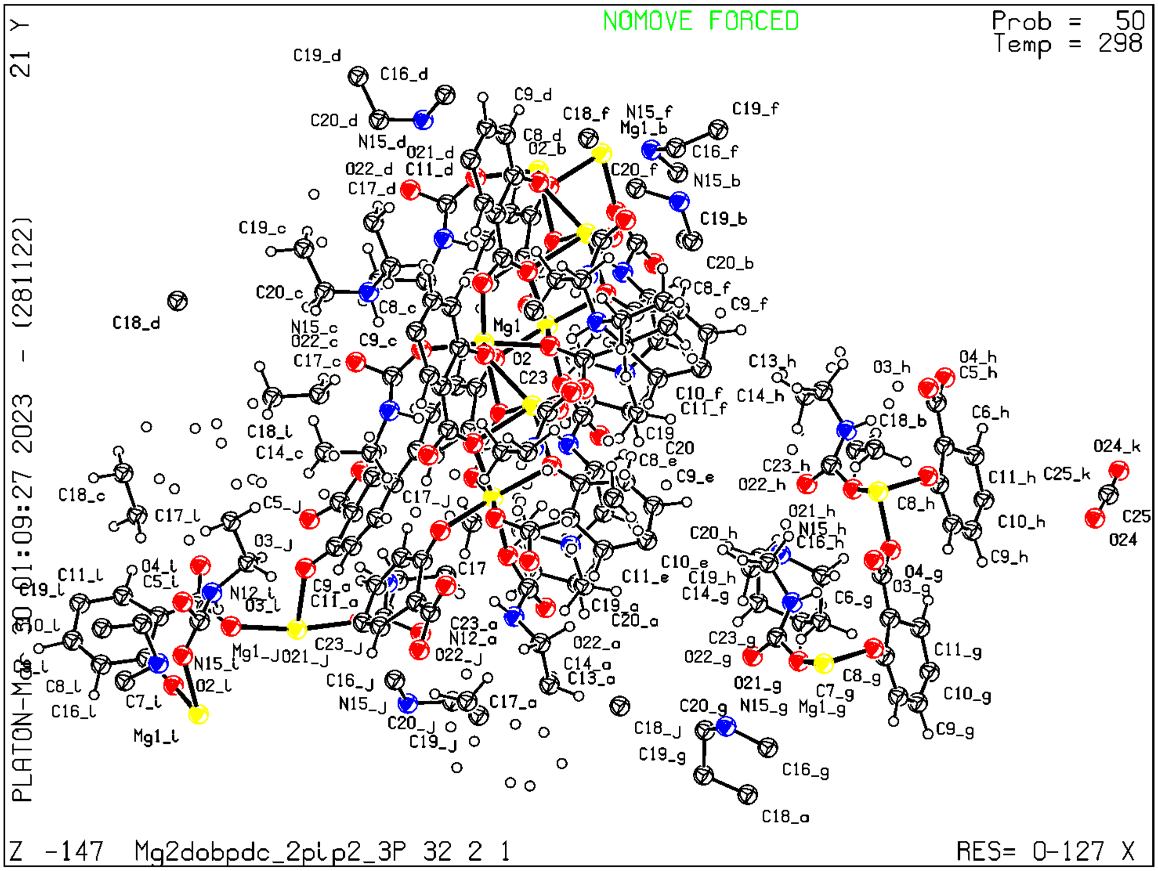

Supplement: Supplementary file 2 — ja3c13381_si_002.zip [file ja3c13381_si_002.zip › Supporting Files/checkcif_CO2-pip2-Mg2dobpdc.pdf]
